# A Cohesin-Independent Role for NIPBL at Promoters Provides Insights in CdLS

**DOI:** 10.1371/journal.pgen.1004153

**Published:** 2014-02-13

**Authors:** Jessica Zuin, Vedran Franke, Wilfred F. J. van IJcken, Antoine van der Sloot, Ian D. Krantz, Michael I. J. A. van der Reijden, Ryuichiro Nakato, Boris Lenhard, Kerstin S. Wendt

**Affiliations:** 1 Department of Cell Biology, Erasmus MC, Rotterdam, Netherlands; 2 Computational Biology Unit, Uni Computing, Uni Research AS, Bergen, Norway; 3 Center for Biomics, Erasmus MC, Rotterdam, Netherlands; 4 The Children's Hospital of Philadelphia, University of Pennsylvania School of Medicine, Philadelphia, Pennsylvania, United States of America; 5 Laboratory of Genome Structure and Function, Institute of Molecular and Cellular Biosciences, The University of Tokyo, Tokyo, Japan; 6 Department of Biology, University of Bergen, Bergen, Norway; Stanford University School of Medicine, United States of America

## Abstract

The cohesin complex is crucial for chromosome segregation during mitosis and has recently also been implicated in transcriptional regulation and chromatin architecture. The NIPBL protein is required for the loading of cohesin onto chromatin, but how and where cohesin is loaded in vertebrate cells is unclear. Heterozygous mutations of NIPBL were found in 50% of the cases of Cornelia de Lange Syndrome (CdLS), a human developmental syndrome with a complex phenotype. However, no defects in the mitotic function of cohesin have been observed so far and the links between NIPBL mutations and the observed developmental defects are unclear. We show that NIPBL binds to chromatin in somatic cells with a different timing than cohesin. Further, we observe that high-affinity NIPBL binding sites localize to different regions than cohesin and almost exclusively to the promoters of active genes. NIPBL or cohesin knockdown reduce transcription of these genes differently, suggesting a cohesin-independent role of NIPBL for transcription. Motif analysis and comparison to published data show that NIPBL co-localizes with a specific set of other transcription factors. In cells derived from CdLS patients NIPBL binding levels are reduced and several of the NIPBL-bound genes have previously been observed to be mis-expressed in CdLS. In summary, our observations indicate that NIPBL mutations might cause developmental defects in different ways. First, defects of NIPBL might lead to cohesin-loading defects and thereby alter gene expression and second, NIPBL deficiency might affect genes directly via its role at the respective promoters.

## Author Summary

The cohesin complex is crucial for chromosome segregation during cell divisions but was recently also implicated in transcriptional regulation and chromatin architecture. Cohesin's binding to chromatin depends on NIPBL, a factor that was found to be mutated in 50% of the cases of the human developmental disorder Cornelia de Lange Syndrome (CdLS). To understand the role of NIPBL for cohesin, we need to know when and where the cohesin is loaded onto DNA. Our experiments have identified high-affinity NIPBL binding sites in different cells lines which do not overlap with cohesin-binding, but colocalize with specific transcription factors at active promoters. The activity of the respective genes depends on NIPBL but not cohesin. This is in contrast with other published data showing colocalization of NIPBL and cohesin, and we reveal the existence of different types of NIPBL binding sites that are detected differently by the antibodies used in the different studies. Our observations reveal a dual role for NIPBL in cohesin loading and as potential transcription co-factor, which yields novel insights into how NIPBL defects could cause Cornelia de Lange Syndrome since NIPBL mutations might directly influence developmentally important genes.

## Introduction

Genomes need to be stably inherited over numerous cell generations. For each cell division the genetic information has to be replicated, the copies identified and then equally distributed between daughter cells. This process crucially depends on the cohesin complex, consisting of the core subunits SMC3, SMC1A, RAD21, SA1/STAG1 or SA2/STAG2 and several transiently associated regulatory proteins (reviewed in [1]). Cohesin tethers two sister chromatids together from S-phase on, allowing for their proper segregation in mitosis. Furthermore, cohesin is important for DNA damage repair (for review see [2]), for chromatin insulation in cooperation with the chromatin insulator protein CCCTC-binding factor (CTCF) [3]–[5], for chromosomal long-range interactions [6]–[8], and for development [9]–[12]. The latter functions implicate cohesin in regulating gene expression; indeed, a large number of genes are misregulated after cohesin depletion [3], [13].

How exactly cohesin associates with DNA is not understood, since none of the subunits binds directly to DNA. Rather, cohesin is hypothesized to bind to DNA by embracing the DNA strands with a “protein ring” formed by the core subunits [14], [15].

Cohesin's binding to chromatin is tightly regulated throughout the cell cycle. To enable chromosome segregation it is removed from chromosomes during mitosis. A prophase pathway depending on WAPL and specific phosphorylation of cohesin subunits dissociates cohesin from chromosome arms. The remaining cohesin is removed by proteolytic cleavage of the RAD21 subunit at anaphase onset (reviewed in [1]). Cohesin re-associates with chromatin at the G1-S-phase transition in yeast but in vertebrates already earlier during G1 phase.

The chromosomal localization of cohesin is determined by several factors. First, the cohesin loading factors NIPBL (also known as IDN3 or Delangin; Nipped-B, *Drosophila melanogaster*; Scc2, *Saccharomyces cerevisiae)* and MAU2 (also KIAA0892; Scc4 in *Saccharomyces cerevisiae*) are crucial for the re-loading of cohesin in G1-phase after its complete dissociation from chromatin during mitosis (reviewed in [1]). In yeast, it has been shown that cohesin associates first with Scc2 binding sites and then relocalizes to different positions [16], [17]. In *Drosophila melanogaster* cohesin colocalizes with NIPBL to actively transcribed genes [18] and in mouse ES cells a subset of cohesin binding sites was described to colocalize with NIPBL and the mediator complex [13]. Second, factors co-localizing with cohesin on chromatin such as CTCF [3] and Estrogen receptor [19] determine where cohesin is positioned.

Mutations in NIPBL and cohesin subunits, have been linked to the “Cohesinopathy” Cornelia de Lange syndrome (CdLS, OMIM #122470, #300590 and #610759). This dominant, genetically heterogeneous developmental disorder has a high degree of variability in its clinical presentation with multiple organ systems affected. It is estimated to occur in 1∶60000 to 1∶45000 live births. Characteristic features include craniofacial anomalies, growth retardation, intellectual disability, upper limb defects, hirsutism, and involvement of the gastrointestinal and other visceral organ systems [16]. Clinically, CdLS phenotypes can range from very mildly affected (no structural abnormalities, minor intellectual disability) to severely affected (upper limb defects, severe intellectual disability). Heterozygous mutations of NIPBL, ranging from nonsense and frameshift mutations to truncation mutations, have been found in 50% of CdLS patients and mutations of the cohesin subunits SMC1A, and SMC3 were found in another 5% (reviewed in [17]). Observations in patients and mouse models show that in cells with heterozygous NIPBL mutations the NIPBL transcript levels are only reduced by ∼30% due to an increased expression from the intact allele [18], [19]. A clinical phenotype is observed with a modest 15% reduction in expression [20]. This indicates that NIPBL expression levels are tightly regulated and are critical for cells. Defects in cohesin-dependent chromosome cohesion were not observed at this level of NIPBL reduction in CdLS patients or any model systems [19], [21]. However, a reduction of cohesin binding sites was observed in cells derived from CdLS patients, which was most obvious in close proximity to genes [18]. This suggested that the clinical features of CdLS are the collective outcomes of changes in the expression level of multiple genes during development.

NIPBL has already been linked to gene regulation. In Drosophila, NIPBL was found to facilitate the activation of the *cut* and *Ultrabithorax* genes by remote enhancers. In the case of the *cut* gene, NIPBL facilitates its long-range activation while cohesin has an inhibitory effect on *cut* expression [22]. Further, human NIPBL was already shown to bind histone deacetylases (HDAC1, HDAC3) [23] and heterochromatin protein 1 (HP1) [24].

These observations implied a “dual role” for NIPBL, in loading cohesin and in gene regulation. It is not known whether these two functions are independent of each other, or if NIPBL mediates gene regulation via loading of cohesin onto DNA.

In this study we have aimed to determine when and where NIPBL binds to chromatin to determine where cohesin is initially loaded. Furthermore we wanted to elucidate whether the position of NIPBL binding in the genome accounts for the altered gene expression patterns observed in CdLS patients carrying NIPBL mutations [18].

## Results

### Consecutive loading of NIPBL, CTCF and cohesin

**Figure 1:**
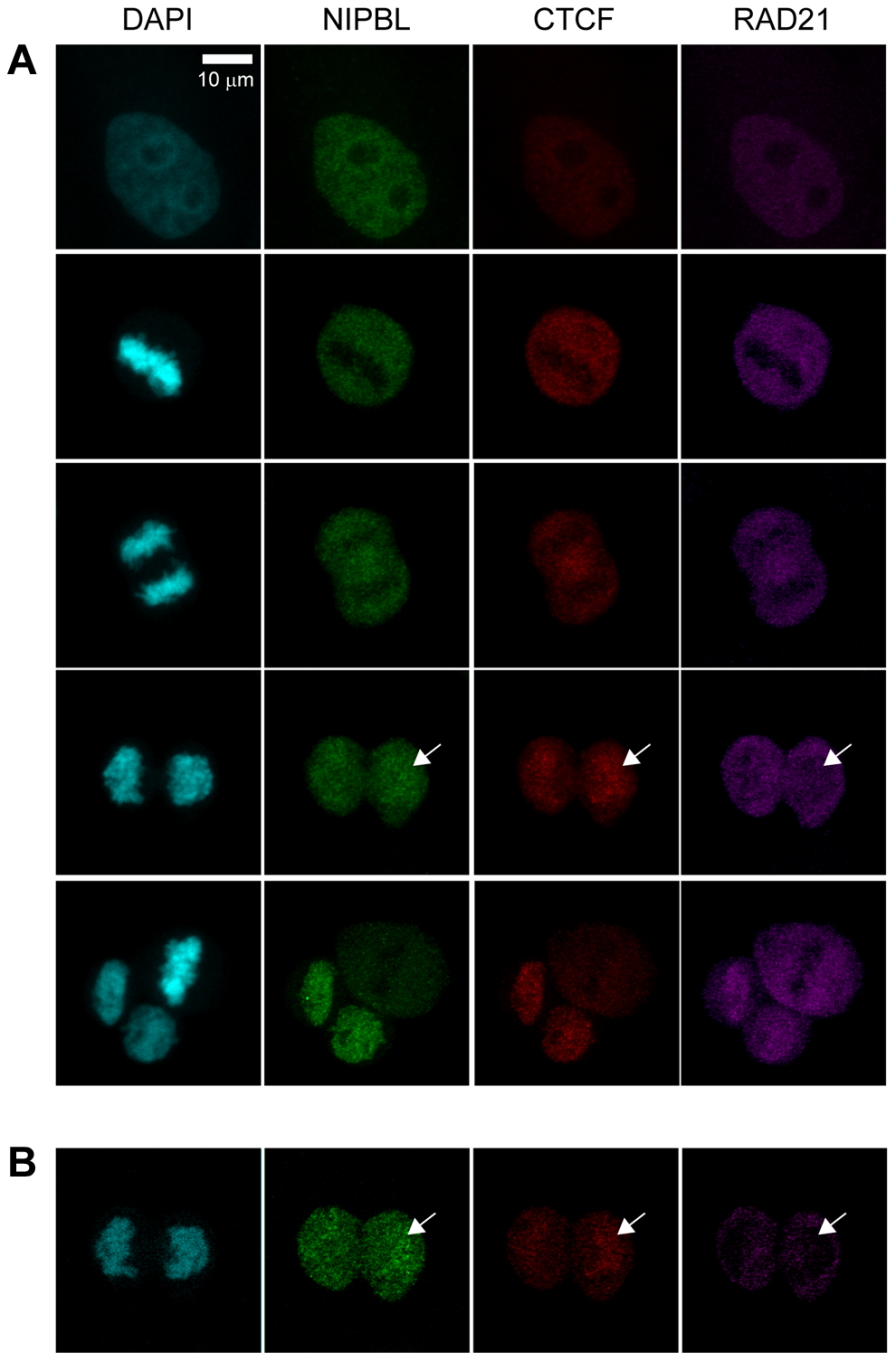
Chromatin association of NIPBL, cohesin and CTCF during exit from mitosis. **A** To address the association of cohesin, CTCF and NIPBL with chromatin during end of mitosis HeLa cells were fixed with PFA and stained with antibodies against CTCF (CTCF#1), the cohesin subunit RAD21 and NIPBL (NIPBL#2). Image stacks were taken with a confocal microscope and a Z-projection generated with Image J. Cells in interphase and different stages of mitosis are shown, from top to bottom: interphase, metaphase, late anaphase, telophase, completed cytokinesis together with a metaphase. **B** One image slice (100 µm) of the telophase images in (**A**) is shown to highlight the lack of cohesin signal on chromatin while NIPBL and CTCF are already present.

To gain insight into the cohesin loading mechanism it is crucial to understand when cohesin interacts with these factors during the loading process. We have therefore compared the timing of the chromatin-localization of cohesin with that of NIPBL and CTCF. Mitotic HeLa cells were fixed with paraformaldehyde (PFA) and immunostained with antibodies specific for CTCF, NIPBL and the cohesin subunits RAD21 and SA2/STAG2 (Fig. 1; Suppl. Fig. S1B, C). Specificity of the antibodies was demonstrated by immunostaining of siRNA-depleted cells (Suppl. Fig. S1A). It was then determined at which stage the signals of these proteins appeared on chromatin during the exit from mitosis (Fig. 1). These results were also correlated with the reassembly of the nuclear envelope in HeLa cells expressing Lamin B-EGFP. Similar to cohesin we find the signals of NIPBL and CTCF to be largely excluded from metaphase chromosomes. However to our surprise both NIPBL and CTCF signals appear on chromatin at an earlier stage of the mitotic exit than cohesin (Fig. 1), actually before the nuclear envelope is reassembled as shown by comparison to Lamin B signals (Suppl. Fig. S1B). Therefore NIPBL and CTCF are already present on chromatin, before the cohesin complex begins to re-associate with chromatin. This suggests that NIPBL binds first to chromatin and subsequently recruits cohesin. The fact that CTCF associates with chromatin before cohesin enforces our earlier observation that cohesin is dispensable for CTCF localization on chromatin [3].

### NIPBL localizes in somatic cells independently of cohesin

To analyze the genomic localization of NIPBL binding sites relative to cohesin and CTCF, we selected the NIPBL antibody (referred to as NIPBL#1) that performs best in human cells (Suppl. Fig. S2) and performed ChIP-sequencing for NIPBL, cohesin and CTCF using HB2 cells (1-7HB2) [25] enriched in G1 phase (Suppl. Fig. S3A) and for NIPBL in lymphoblastoid cells (LCL; B-cell population immortalised by EBV-transformation) derived from a normal control (N5) and CdLS patients (PT1, PT9).

Furthermore, we have determined the transcriptional activity by RNA-sequencing, and identified active transcription start sites in HB2 cells by ChIP-sequencing of RNA Polymerase II (RNA Pol II). ChIP for NIPBL, SMC3, CTCF and RNA Pol II was performed as described [3], but for SMC1A ChIP a SDS-free protocol was used to maximize the ChIP-efficiency [26].

To prove the specificity of the identified peaks for NIPBL we have depleted NIPBL by RNAi and observed greatly reduced ChIP-qPCR signals for the analysed sites (Suppl. Fig. S4 A–C).

Using the criteria described in the Materials and Methods section, we identified 1138 NIPBL sites, 35668 CTCF sites, 22572 SMC3 sites and 29441 SMC1A sites in HB2 cells and between 1600 and 2000 NIPBL sites in lymphoblastoid cells (LCL). The data from the different LCL's and the conclusions for CdLS are discussed in detail in a later section.

**Figure 2:**
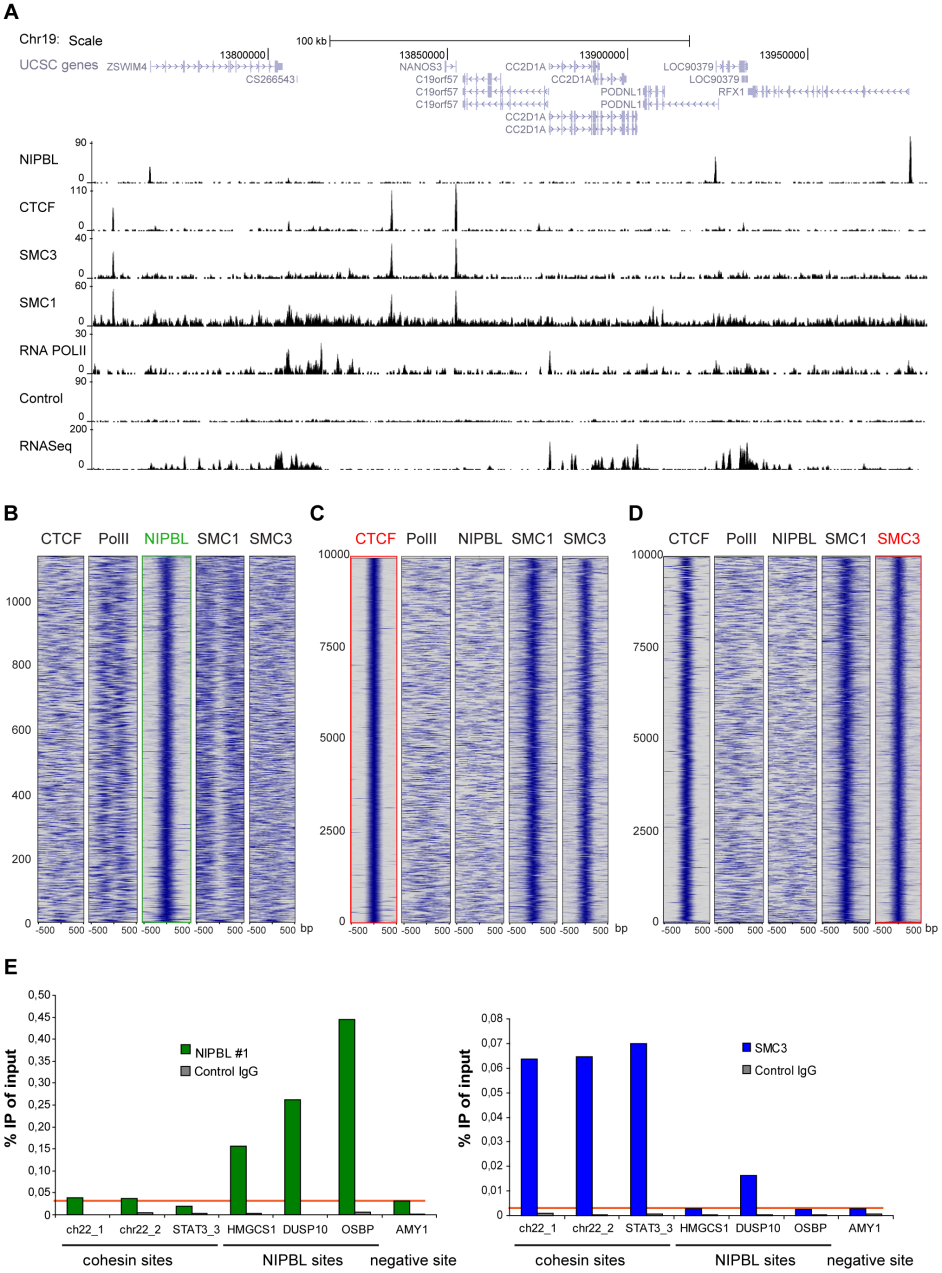
Binding of NIPBL, cohesin and CTCF in the human genome. **A** Genomic binding of NIPBL, CTCF and the cohesin subunits SMC3 and SMC1A in the breast endothelial cell line HB2 at a selected region of chromosome 19 as determined by ChIP-sequencing. The RNA Pol II binding profile, the control ChIP and the RNA-sequencing data from these cells are also shown. **B–D** Heatmaps showing the ChIP signal intensity of the indicated ChIP-sequencing experiments in a window of +/−500 bp around all NIPBL peaks (**B**) as well as the top 10000 CTCF (**C**) and SMC3 (**D**) peaks. Cohesin (SMC3, SMC1A) and CTCF binding does not correlate with NIPBL binding events. RNA Pol II signals are found near NIPBL, consistent with the localization of NIPBL at promoters. Cohesin binding events correlate well between SMC3 and SMC1a and with CTCF. Peaks are ranked by size with the strongest peaks at the bottom of the graph. **E** ChIP was performed with NIPBL#1, SMC3 and control antibodies from HB2 cells and analyzed by qPCR with primers specific for cohesin, NIPBL and a negative (*AMY*) sites. NIPBL ChIP signals on cohesin sites are at background level (red horizontal line). Only the DUSP10 site is higher than the background in the SMC3 ChIP, very likely due to a CTCF/cohesin site close to the NIPBL site. All experiments were at least performed three times and one representative example is shown.

Surprisingly, in HB2 cells the NIPBL binding sites do not overlap with cohesin or CTCF binding sites (Fig. 2A). Heatmaps centred on NIPBL (Fig. 2B), cohesin or CTCF binding sites (Fig. 2C, D), show no overlap of cohesin or CTCF signals with NIPBL sites. As expected, there was a high correlation between cohesin and CTCF signals. The absence of overlapping NIPBL and cohesin sites was confirmed by qPCR analysis of several NIPBL and cohesin binding sites in SMC3 and NIPBL ChIP experiments, where we observe only background levels of NIPBL binding on cohesin sites and vice versa (Fig. 2E).

Cohesin binding was previously observed on centromeric repeats and Alu elements [27]–[29], therefore we also analysed sequencing reads mapping uniquely to repeat sequences (Table S8). NIPBL ChIP highly enriches rRNA repeats (13 fold), in particular the large (LSU, 15 fold enriched) and small subunit (SSU, 14 fold enriched) repeat families but not at the repeat classes described for cohesin. rRNA repeats are pseudogenes of unknown function distributed all over the human genome [30]. In total we observe NIPBL at 20 out of 467 known LSU/SSU regions (Hg19 assembly of the human genome) and by ChIP-qPCR with primers specific for LSU and SSU repeats we confirmed NIPBL-binding to four of five LSU repeat regions and one of three SSU regions (Suppl. Fig. S5A).

The missing colocalization between NIPBL and cohesin is in contrast with observations in mouse embryonic stem cells (mESC) [13]. To address this we critically reviewed the ChIP-sequencing data analysis from Kagey et al., the ChIP protocols used and the different antibodies, NIPBL#1 from our study and NIPBL#6 used by Kagey et al.. Our review of the ChIP-seq data analysis from Kagey et al. confirmed their general finding that cohesin and NIPBL ChIP signals overlap, although we did not find such a colocalization of NIPBL and cohesin in our study. Further, we compared the different ChIP protocols by performing ChIP from mESC using both protocols and both antibodies (Suppl. Fig. S5B, C). We observe a better ChIP/IgG-control ratio using our protocol, which includes a more stringent washing of the beads (Suppl. Fig. S5C). For three NIPBL sites at promoters (*Nanog*, *Lefty*, *Oct4*), identified by Kagey et al. in mESC [13], both antibodies perform weakly but equally well, independent of the ChIP protocol (Suppl. Fig. S5B, C). To demonstrate once more the specificity of both antibodies for NIPBL, we have performed ChIP with both antibodies from control mESC and mESC derived from a *Nipbl+/−* mouse embryo (Suppl. Fig. S5D) (S. Goldberg, F. Grosveld unpublished data) and observe with both antibodies a 20–40% decreased Nipbl binding at all sites (Suppl. Fig. S5E). This is consistent with previous reports on *Nipbl+/−* mESC that heterozygous knockout cells still have 70% of wild-type Nipbl mRNA levels [19].

However, on the NIPBL binding sites that we find to be conserved between human HB2 cells and mES (*Tiam1*, *Ankhd1*, *Sp1*), the ChIP is strikingly better enriched for NIPBL#1 than NIPBL#6 in both cell types (Suppl. Fig. S5B, C, F). Therefore, different chromatin morphologies between pluripotent and differentiated cells do not account for the different binding patterns.

We conclude from these results that there are two different types of NIPBL binding sites. The NIPBL#1 antibodies highly enrich for a set of “major sites” that localize at promoters and do not overlap with cohesin. The NIPBL#6 and NIPBL#1 antibodies both detect a set of low-enriched sites (“minor sites”, low ChIP/seq signals) which overlap with cohesin binding sites.

### NIPBL binds to active promoters, together with a distinct set of transcription factors

**Figure 3:**
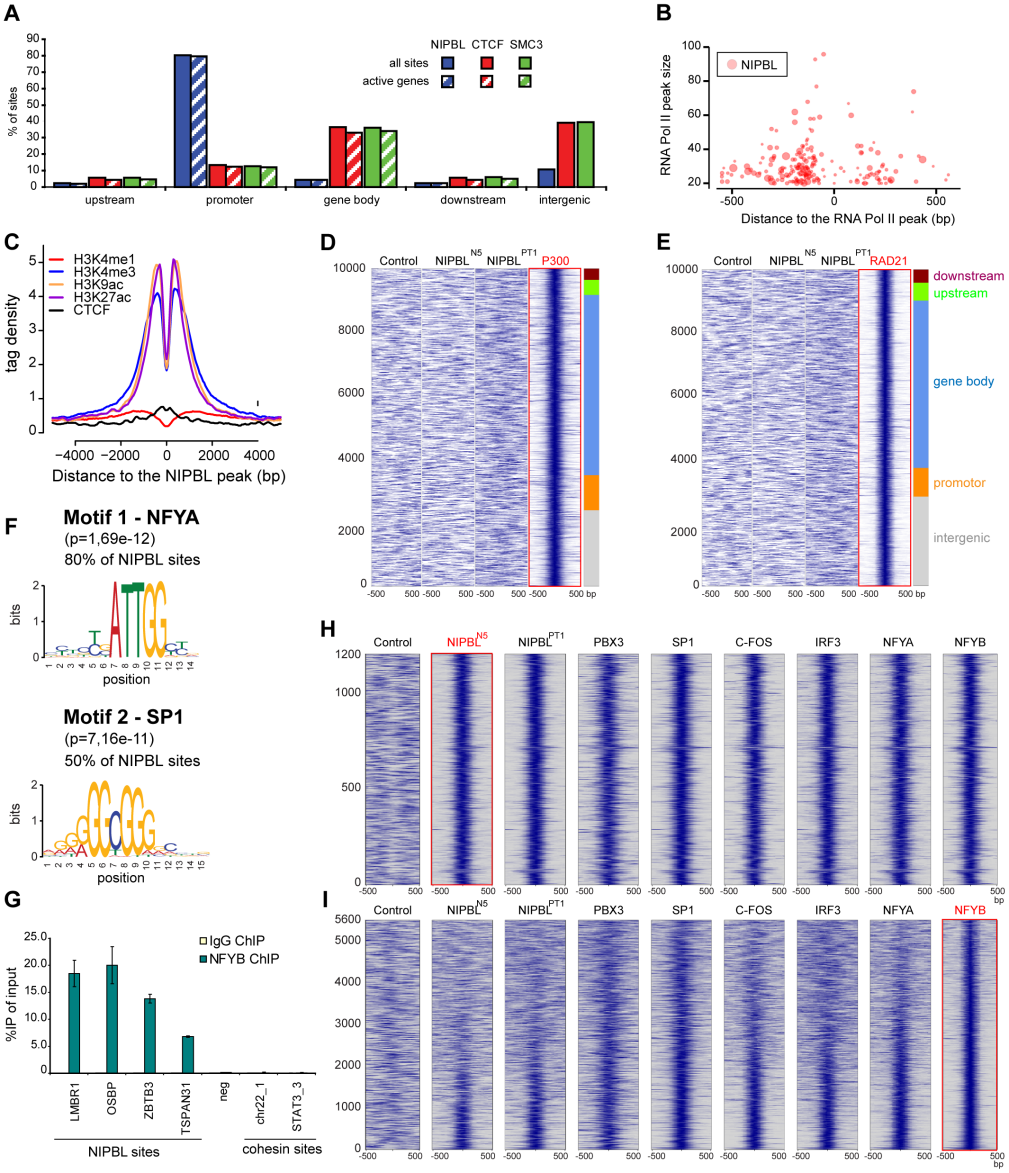
NIPBL binds to active promoters together with other transcription factors. **A** Binding of NIPBL, CTCF and cohesin (SMC3) relative to active genes in HB2 cells. The different regions were defined as follows; upstream: −5 kbp to −1 kbp from transcription start sites; promoter: 1 kbp upstream and downstream from TSS; gene body: +1 kbp from TSS until end of the coding sequence; downstream: end of the coding sequence - +5 kbp (See also Table S2). **B** Bubble plot representation of NIPBL binding around RNA Pol II peaks in HB2 cells. The x-axis denotes the position of NIPBL respective to the closest RNA Pol II peak and the y-axis the strength of the RNA Pol II peak. Bubble size indicates the strength of the NIPBL peak. NIPBL binds 100–250 bp around RNA Pol II peaks, preferentially upstream, which is consistent with binding to active promoters. **C** NIPBL binding in the control LCL's (N5) was compared with localization of histone modifications and CTCF in the lymphoblastoid cell line GM12878 [31]. The plot is centred on the NIPBL peaks and the y-axis displays the signal intensity of the respective histone modification and CTCF in GM12878 cells. **D** Heatmap correlating the P300 ChIP signals +/−500 bp around P300 binding sites observed in GM12878 cells with the sequencing reads obtained for the control and for NIPBL ChIP in control (N5) and patient cells (PT9). The plot is centred on the 10000 strongest P300 peaks clustered into different genomic regions as in (A). **E** Identical heatmaps generated for the RAD21 peaks observed in GM12878 cells. **F** Consensus motif derived de-novo from NIPBL binding sites in HB2 cells. The region ±50 bp around the peak maximum was used to determine motifs with MEME [33]. These motifs are nearly identical to the respective motifs of the transcription factors NFYA and SP1, indicating that one or more transcription factors might colocalize with NIPBL. **G** Binding of NFYB to NIPBL sites as discovered by the motif analysis in (D) and the comparison to binding sites of other transcription factors in (E) was confirmed by ChIP-qPCR with anti-NFYB antibodies. **H** Heatmaps comparing +/−500 bp around NIPBL sites observed in LCL's (N5) with ChIP-sequencing data of various transcription factors (GM12878 cells) revealed a subset of transcription factors colocalizing with NIPBL. The heatmaps reveal a strong correlation of PBX3, SP1, C-FOS, IRF3 and NFYA/B with NIPBL sites. **I** Heat maps showing the correlation of the factors in (H) to NFYB sites at GpG island promoters (sites at CpG island promoters ranked according to strength with the strongest signals at the bottom). The strongest correlation with the other factors is visible for the strongest NFYB peaks.

NIPBL “major binding sites” are distributed over the entire genome (repetitive sequences were omitted during the mapping of the reads to the genome) but localize very specifically to the promoter area (+/−1000 bp from transcription start sites) (Fig. 3A). We observe such localization for 912 of 1138 (80%) NIPBL sites in HB2 cells, while only ∼10% of the cohesin and CTCF sites localize to promoters. About 89% of NIPBL-bound promoters are CpG island promoters (Table S4). Analysis of RNA-sequencing data from HB2 cells revealed that >98% of these NIPBL-bound genes are actively transcribed (Fig. 3A and Table S3), indicating a preferential binding of NIPBL to active promoters. Comparison with RNA Pol II binding sites showed that NIPBL preferentially binds 100–200 nucleotides upstream of RNA Pol II (Fig. 3B). This correlation is also visible as bimodal distribution of the RNA Pol II signal since orientation of transcription was not considered in this plot (Fig. 2B).

To analyse the properties of NIPBL binding sites further, we used the NIPBL binding sites observed in the control LCL's (N5), since a large number of data for histone modifications and transcription factors is available for lymphoblastoid cells like GM12878 from earlier publications [31] and ENCODE [32].

Comparing the pattern of different histone modifications around NIPBL sites, we observed that the sites are flanked by histone marks linked to active promoters and enhancers (H3K4me3, H3K27ac and H3K9ac) (Fig. 3C). However, the H3K4me1 mark, characteristic for enhancers, does not show enrichment (Fig. 3C). NIPBL itself apparently resides in nucleosome-free areas.

The missing enhancer-specific histone mark is in contrast with observations in mouse ES cells showing a colocalization of NIPBL with enhancers and cohesin [13]. Therefore we also compared the NIPBL binding with the enhancer marker p300 (Fig. 3D) and the cohesin subunit RAD21 (Fig. 3E) and again observed no correlation.

Motif analysis of NIPBL binding sites in HB2 cells and LCL's using MEME [33] reveals that the motifs for the transcription factor NFYA (subunit of the NF-Y complex) are present at 80% of NIPBL sites and for SP1 at 50% of the sites (Fig. 3F). NF-Y binds the CCAAT box, which correlates well with the presence of CpG islands at promoters; also, a connection between NF-Y and SP1 has often been reported with presence of both motifs at the same promoter. To test whether the presence of the NFYA motif is correlated to the CpG-island promoter or a genuine property of the NIPBL-bound promoters we analyzed NIPBL-bound CpG island promoters versus randomly selected CpG island promoters and observe a statistical significant preference (Fisher test, p<0.001) of NFYA for NIPBL-bound CpG island promoters. ChIP with anti NFYB antibodies from HeLa cells confirms binding of the NF-Y complex to NIPBL binding sites determined above (Fig. 3G).

To investigate whether other transcription factors colocalize specifically with NIPBL we compared the NIPBL sites in LCL's with available ChIP-sequencing data for transcription factors for GM12878 cells collected by ENCODE [32]. Specifically, we analyzed in total 66 binding profiles and generated heat maps covering +/−500 bp around NIPBL binding sites conserved in lymphoblastoid cells. By visual inspection of the maps we identified five transcription factors present on NIPBL sites: NFYA/NFYB and SP1, which is consistent with the presence of the motif, as well as PBX3, C-FOS and IRF3 (Fig. 3H). The heatmaps displaying the signals of the other transcription factors on NIPBL binding sites show a very good correlation between all five factors. When the signals are plotted respective to NFYB sites sorted according to peak intensity, it shows that NIPBL and several other factors overlap only with the strongest NFY peaks (Fig. 3I).

### NIPBL is important for gene activity

NIPBL-bound genes in HB2 cells were analyzed using IPA (Ingenuity Systems, www.ingenuity.com) and found to be linked to diverse cellular functions, such as cell cycle control, gene expression, cell death, RNA post-translational modification and control of cellular growth and proliferation (Table S5). Out of 1118 NIPBL-bound protein-coding genes, 122 (11%) were classified as transcription factors by Vaquerizas et al. 2009 [34], which is not a statistically significant enrichment compared to the number of transcription factors in lists with randomly selected genes, but indicates that important developmental genes might depend on NIPBL. Examples are *SP1*, *SP2*, *SP3*, *BBX* and *STAT3*, all important transcription factors for development and NIPBL binding at their promoters could be important for their appropriate expression.

**Figure 4:**
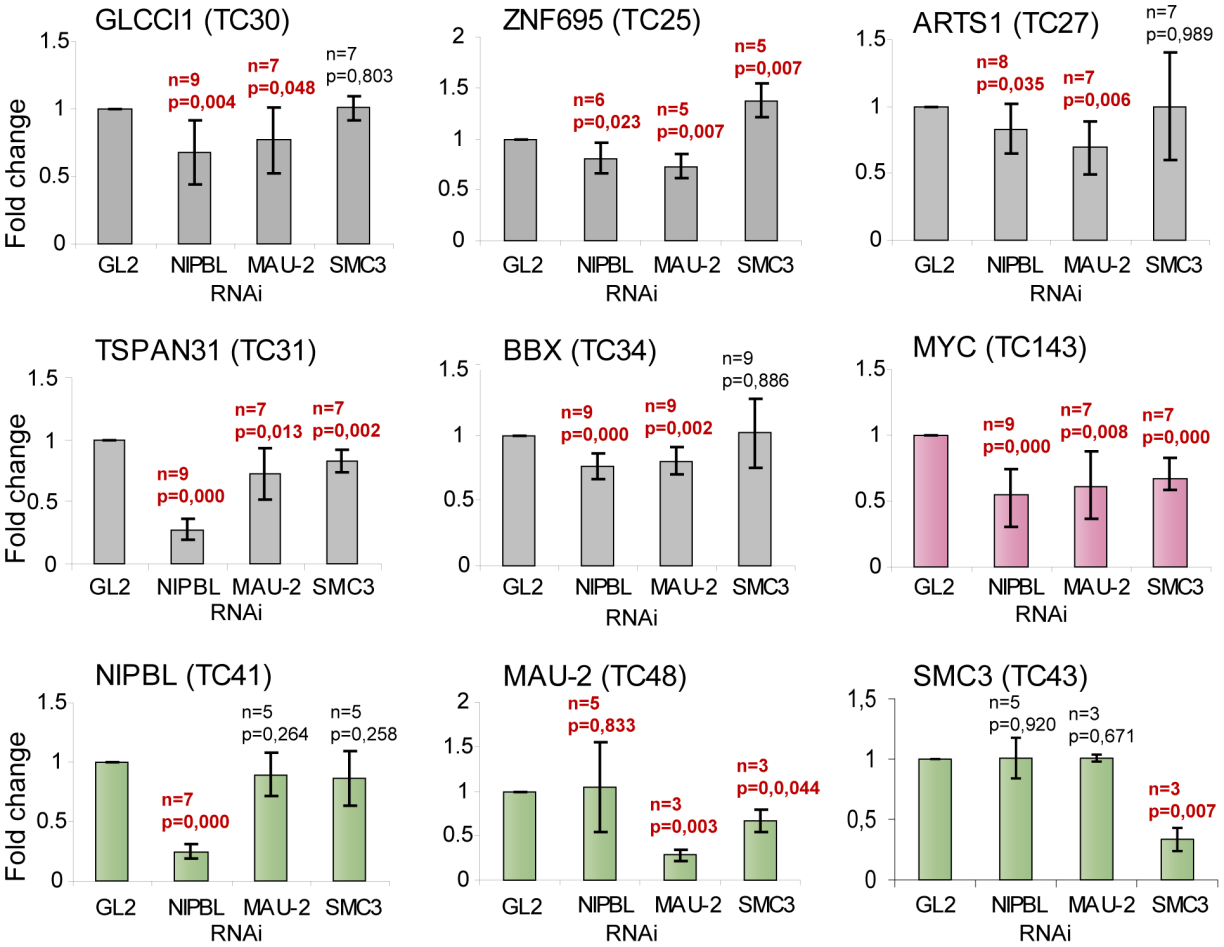
NIPBL is important to maintain gene activity. Transcript levels of genes with NIPBL-bound promoters and no cohesin sites close to the gene (*GLCCI1*, *BBX*, *TSPAN31*, *ARTS-1* and *ZNF695*) and the cohesin-regulated *MYC* gene were analyzed by RT-PCR/qPCR after RNAi depletion of NIPBL, MAU2 or SMC3 in HB2 cells. The cells were synchronized in G2 phase and the transcript levels are normalized against the housekeeping gene *NAD*. Transcripts of NIPBL, MAU2 and SMC3 were also analyzed to exclude that NIPBL affects transcription of MAU2 and SMC3 and vice versa. All three genes serve also as negative control genes without NIPBL binding site at the promoter, although MAU2 and SMC3 have intronic cohesin binding sites. P-values were determined using Students test using between 3 and 9 independent biological replicates. The p-value and number of replicates is indicated for each graph. Values that are significantly different (P-value<0.05) from control RNAi are highlighted in red. (error bars ± s.d.).

To address whether NIPBL is important for the active transcription of the associated genes, we selected functionally different genes with conserved NIPBL binding at the promoter, but no cohesin binding site close to or on the gene, and asked whether their transcription changes in HB2 cells after knockdown of NIPBL, MAU2 or SMC3. To avoid problems in cell division due to impaired sister chromatid cohesion, we synchronized cells in G2 phase during the siRNA treatment (Suppl. Fig. S3B). Out of the seven initially selected genes, five showed statistically significant changes after NIPBL RNAi depletion: *GLCCI1*, a glucocorticoid inducible transcript; *TSPAN31*, encoding a transmembrane protein involved in signal transduction and growth-regulation; *BBX*, encoding a HMG-BOX transcription factor; *ZNF695*, an uncharacterized zinc-finger protein and *ARTS-1/ERAP1*, an endoplasmic reticulum aminopeptidase. Transcript levels were analyzed by RT-PCR and qPCR and normalized against the housekeeping gene *NAD*. Depletion of NIPBL and also of MAU2 leads to a statistically significant (*t*-test, P-values<0.05) decrease of gene expression levels of the candidate genes (Fig. 4), indicating that NIPBL and MAU2 dosage are important for maintaining expression levels. The depletion of SMC3 did not significantly reduce the expression of these transcripts, although the expression of the known cohesin-regulated *MYC* gene [35] was reduced. This indicates that the changes in expression as a result of NIPBL depletion are not the indirect result of reduced cohesin binding and cohesin's role for transcription.

### Insights into Cornelia de Lange Syndrome (CdLS)

**Figure 5:**
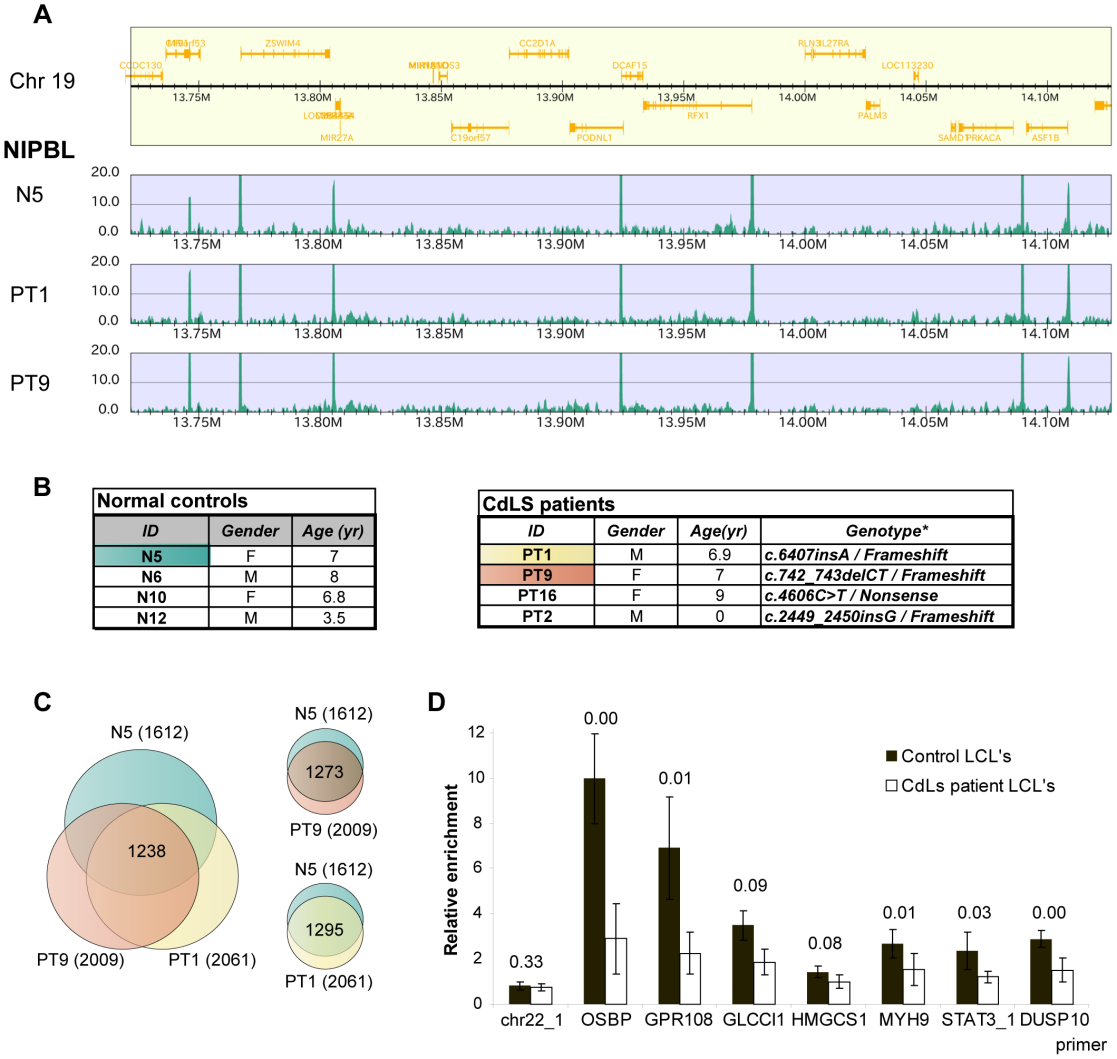
Position of NIPBL sites is conserved but the occupancy is reduced in CdLS. **A** NIPBL ChIP-sequencing data of a region of chromosome 19 showing that NIPBL sites are conserved between CdLS patient cells and the control (M – Megabases). **B** CdLS patient and control cell lines used in this study. The cell lines highlighted were used for ChIP-sequencing. The tables were derived from [18]. Nucleotide numbering refers to the *NIPBL B* isoform cDNA sequence with GeneBank accession number NM_015384 and starting at the +1 position of the translation initiation codon. **C** Venn diagrams indicating the number of NIPBL binding sites observed in the different LCL's and also the sites consistently called in all three lines. The majority of binding sites is conserved, although each cell line displays cell-line specific sites. **D** NIPBL binding is reduced in LCL's derived from CdLS patients. NIPBL ChIP was performed for four patient-derived cell lines and four age and gender-matched controls and qPCR analysis was performed for seven NIPBL binding sites and one cohesin site. The enrichment compared to control IgG ChIP was calculated. The data for the individual cell lines are displayed in Suppl. Fig. S6. Here we present the average relative enrichment for all control and patient-derived lines, p-values derived with a Student test are indicated above the respective columns.

Mutations in the *NIPBL* gene have been identified in approximately 50% of CdLS patients. Our discovery that NIPBL binds to active promoters prompted us to identify the major NIPBL binding sites in lymphoblastoid cells (LCL's) derived from blood samples of severely affected CdLS patients with NIPBL truncation mutations and normal controls (Fig. 5A, B).

Using the NIPBL#1 antibody we detected 1612 major NIPBL sites in the control (N5) and 2061/2009 sites in the patient-derived lines (PT1/PT9), with 1295 sites overlapping between N5/PT1 and 1273 sites between N5/PT9. In summary 80% of the sites in the control N5 are also found in PT1 and PT9 (Fig. 5C). The majority (74%) of the sites observed in HB2 cells is consistent with these conserved sites, indicating conservation between different tissues. Consistent with our observations in HB2 cells, most NIPBL binding sites in the LCL's localize to the 5′ ends of genes and are enriched for the motifs of the transcription factors NF-Y and/or SP1. Gene ontology analysis of the LCL NIPBL-bound genes showed similar classes of genes as for HB2 cell, but no cell type-specific functions such as immune response.

Although expected from patient-derived cell lines with NIPBL haploinsufficiency, we did not observe significant differences in peak number or peak intensity between controls and patient-derived LCL's. This is explained by the rather small differences of NIPBL protein levels between CdLS patients and controls [22] due to increased transcription from the intact allele. The ChIP-sequencing method is not quantitative and therefore small changes of NIPBL levels might not be reflected by peak intensity. To address this we performed NIPBL ChIP-qPCR from four control cell lines and four CdLS patient cell lines with primers for seven NIPBL binding sites and one cohesin binding site (negative control). QPCR revealed a reduction of the NIPBL signal between the control and patient-derived cell lines (Fig. 5D; Suppl. Fig. S6), but also variations among individual control- and patient-derived cell lines. In general, strong NIPBL binding sites (*OSBP*, *GPR108*) seem to be more reduced than weaker binding sites.

The position of NIPBL at promoters could be important for the emergence of the developmental defects seen in CdLS cases. Therefore we compared NIPBL binding sites with a list of genes found to be differentially expressed between LCL's from CdLS patients and controls [22]. We compared the list of 1501 unique genes (FDR<0.05) found to be differentially expressed between controls and CdLS patients [22] with our list of 1671 genes neighbouring a NIPBL site (+/−2 kb) in the patient-derived LCL's (PT1) and found that 155 (10%) of these genes are differentially expressed (Table S7), a statistically significant number when compared to a random list of genes (Fisher test, p<0.001).

## Discussion

In its best-studied function NIPBL promotes the initial deposition of the cohesin complex onto chromatin, but is dispensable for maintaining the subsequent association of cohesin and chromatin. Rules that regulate the place and time of cohesin loading and targeting to its various functions (sister chromatid cohesion, transcriptional regulation, mediating long-range chromatin interactions and DNA damage repair) are only partly understood. Factors interacting with chromatin-bound cohesin such as the chromatin insulator CTCF [3], [4], [36] and to a smaller extend estrogen receptor alpha (ERa) [37] determine the localization of cohesin, but not its general chromatin binding [3]. They might either direct NIPBL-dependent cohesin loading to their binding sites or capture cohesin complexes that slide along the DNA fibre.

First, we have addressed when cohesin, CTCF and NIPBL associate with chromatin. So far, only very weak and probably transient interactions have been reported between cohesin and NIPBL in the non-chromatin-bound pool of nuclear proteins [38]. If these transient interactions are sufficient for NIPBL to bind cohesin and recruit it onto chromatin, we would expect the proteins to appear on chromatin at the same time after mitosis. The same is true for CTCF. Analysis of cells exiting mitosis by immunofluorescence staining showed that NIPBL, CTCF and cohesin are largely excluded from metaphase chromosomes, as seen before [3]. The signals of NIPBL and CTCF reappear on DNA before the nuclear envelope reassembles; however, cohesin overlaps with chromatin only during or after the nuclear envelope reformation, reinforcing what was previously described by Gerlich et al. [39]. NIPBL and CTCF are therefore already present when cohesin starts to associate with chromatin. This is consistent with cohesin being dispensable for CTCF localization [3]. NIPBL very likely associates first with chromatin and then recruits' cohesin which is subsequently localized by CTCF to the co-occupied binding sites.

Second, we determined the genomic localization of NIPBL by ChIP using a NIPBL-specific antibody (NIPBL#1) form HB2 cells enriched in G1 phase. We observed about 1100 highly enriched NIPBL sites, mostly at active CpG-island promoters but also at several LSU and SSU rRNA repeat regions. However, we do not observe colocalization with cohesin or CTCF. Missing overlap between NIPBL and cohesin was observed before. In yeast, non-overlapping foci were observed for Scc2 (NIPBL ortholog in *S. cerevisiae*) and Scc1 (RAD21 ortholog in *S. cerevisiae*) by immunofluorescence microscopy on spread chromatin [40]. Further, a ChIP-microarray study in budding and fission yeast observed a transient overlap between cohesin and Scc2 in G1 phase cells and a subsequent relocalization of cohesin to more permanent positions between convergently transcribed genes [41]. Another study in yeast confirmed this property of cohesin [42] while a third study observed that colocalization of Scc2 with cohesin persists also after cohesin loading [43]. In *D. melanogaster* the NIPBL ortholog, Nipped-b, was found to colocalize with cohesin and often overlap with RNA polymerase II, decorating entire active transcriptional units [44]. Remarkably, cohesin does not colocalize with CTCF in the fruit fly.

However, a study in mouse embryonic stem cells (mESC) used a different NIPBL antibody (NIPBL#6) and reported that NIPBL occupies enhancers and core promoter regions of transcriptionally active genes which are also bound by cohesin and Mediator, a huge transcriptional co-activator complex [13] (for review see [45]).

Although we observe a similar localization of NIPBL, we did not detect cohesin binding at NIPBL sites, even with relaxed parameters for peak calling and using different ChIP protocols. We have considered that the apparent discrepancies in the binding patterns might arise due to the different ChIP protocols or differences between pluripotent and differentiated cells, but have disproved these hypotheses by ChIP-qPCR experiments using both antibodies (Suppl. Fig. S5). Importantly, we do observe significant differences between the performances of both antibodies. Immunoprecipitation experiments showed that the NIPBL#1 antibodies recognize more bands originating from NIPBL than NIPBL#6 antibodies (Suppl. Fig. S2). The NIPBL#1 antibodies we use show a similar weak enrichment in ChIP-qPCR experiments as observed for the NIPBL#6 antibodies in mESC (Suppl. Fig. S5C). However, the NIPBL sites identified by our study are highly enriched only by the NIPBL#1 antibodies, not by NIPBL#6. We therefore conclude that the NIPBL#1 antibodies very specifically recognize a number of “major” NIPBL binding sites at active promoters where NIPBL localizes independently from cohesin. The striking localization of NIPBL to promoter of active genes suggested that NIPBL may have a direct role for the transcription of the associated genes. We observe that the transcript levels of several NIPBL-bound genes decrease after RNAi depletion of NIPBL and MAU2. An effect on the transcripts by impaired cohesin loading cannot be entirely excluded but we observe that depletion of SMC3 does not have the same effect on the transcripts. Therefore we hypothesize that NIPBL could have a role as transcription factor, independent from its function for cohesin. A differential effect of NIPBL and cohesin has already been observed in the fly. Nipped-b facilitates activation of the *cut* gene, but stromalin/Scc3, the fly orthologs of the SA1/SA2 cohesin subunit, inhibits its activation. A recent study in zebrafish using morpholino knockdown observed a reduced transcription of several genes, including the transcription factors *sox17*, *foxa2* and *sox32*, after NIPBL knockdown but not in smc3 and rad21 morphants [46].

We found that 11% of NIPBL-bound genes are transcription factors according to Vaquerizas et al. 2009 [34]. A number of them are very important during development and can also be found on the list of genes differentially expressed in CdLS, for example *STAT3* and *YBX1* (Table S7). Studies using mouse models show that the absence of some of these factors (STAT3, YBX1) leads to severe developmental defects and embryonic lethality [47]–[49]. NIPBL deficiency could therefore interfere with the proper timing and expression of transcription factors during development.

The observation that NIPBL might be important for gene expression lead us to ask whether NIPBL haploinsufficiency in CdLS can be linked to transcriptional changes observed in these patients. We have determined NIPBL sites in unsynchronized LCL's derived from CdLS patients with NIPBL haploinsufficiency and normal controls. These binding sites are again mostly located at CpG island promoters, not overlapping cohesin or CTCF. The sites are in part conserved between different tissues, indicating that there are constitutive and cell-type specific sites. The positions of the NIPBL binding sites are conserved between the LCL's from patients and controls, but the actual levels of NIPBL binding are reduced in patients with a hypomorphic NIPBL truncation. To link NIPBL sites to differential gene expression we compared NIPBL-bound genes identified in a patient cell line (PT1) with candidate CdLS target genes identified by Liu et al. [18] and observed that a significant percentage (11%, Fischer test p<0.001) of these genes have a NIPBL binding site. When we asked whether NIPBL RNAi affects gene expression (Fig. 4) a subset of these genes was tested and found to be sensitive for NIPBL knockdown. This lead us to the conclusion that a part of the differentially expressed genes in CdLS could be direct targets of NIPBL, and the observed CdLS phenotype could be a cumulative effect of small changes in the transcriptional program of a larger number of genes.

Comparison of NIPBL sites in LCL's with published binding profiles of transcription factors in the lymphoblastoid cell line GM12878 revealed that NIPBL colocalizes with several transcription factors (SP1, NFY, PBX3, c-FOS, IRF3). Pbx3 belongs to the Pbx family of TALE (three amino acid loop extension) class of homeodomain transcription factors, which are implicated in developmental and transcriptional gene regulation in numerous cell types. Pbx3-deficient mice die after birth due to neuronal malfunctions [50]. The factor is important for facial development in mice [51] together with Pbx1 and Pbx2, and a human Pbx3 mutation was linked to heart defects [52]. IRF3 (interferon regulatory factor 3) is an IRF family transcription factor which translocates from the cytoplasm to the nucleus upon activation, where it acts together with CBP/p300 to activate transcription of interferons alpha and beta, as well as other interferon-induced genes (for review see [53]). C-FOS is part of the AP-1 (activator protein 1) transcription factor complex, which also contains the JUN, ATF and MAF proteins. The complex regulates genes involved in cell proliferation, differentiation, apoptosis, angiogenesis and tumour invasion and can have oncogenic but also anti-oncogenic properties depending on cell type or differentiation state [54]. How these factors functionally interact with NIPBL remains to be investigated.

In summary, in this study we have addressed when and where NIPBL binds to the human genome. We have discovered that a subset of very strong “major” NIPBL binding sites preferentially localizes to active promoters, together with a specific set of other transcription factors. NIPBL is important for the activity of the bound genes, suggesting that NIPBL influences transcription in two ways; directly due to its binding to the promoters and indirectly by loading of cohesin complexes which then regulate genes by chromatin insulation and chromosomal long-range interactions. The possibility that NIPBL directly affects expression suggests that NIPBL-deficiency also directly contributes to the complex CdLS phenotype by altering the transcriptional program of developmentally important genes.

## Materials and Methods

### Antibodies

If different antibodies for the same protein were used the antibodies were numbered to clearly identify them in the different experiments.

NIPBL#1 - polyclonal rabbit anti-NIPBL antibody raised against residues 2598–2825 of the *X. laevis* Scc2-1B, purified using the epitope used for immunization (133M).

NIPBL#2 – polyclonal rabbit anti-NIPBL antibody raised against residues 787–1164 of X. laevis Scc-1B, purified using the epitope used for immunization (114M). Generation and characterisation of the NIPBL #1 and NIPBL #2 antibodies have been published already [38].

NIPPBL#3 – monoclonal rat anti-NIPBL, isoform A (long isoform) NP_597677 (Absea, China, 010702F01 clone KT54)

NIPPBL#4 – monoclonal rat anti-NIPBL, isoform B (short isoform) NP_056199 (Absea, China, 010516H10 clone KT55)

NIPPBL#5 - polyclonal rabbit anti-NIPBL antibody raised against a region between amino acid residues 550 and 600 of human NIPBL (Bethyl Laboratories A301-778A)

NIPPBL#6 - polyclonal rabbit anti-NIPBL antibody raised against a region between amino acid residues 1025 and 1075 of human NIPBL (Bethyl Laboratories A301-779A)

CTCF#1 –monoclonal mouse anti-CTCF (BD 612149)

CTCF#2 – polyclonal rabbit anti-CTCF antiserum (Millipore 07-729)

SA2 – monoclonal rat anti-SA2(STAG2) antibody (Frank Sleutels and Niels Galjart)

SMC1A#1 - polyclonal rabbit anti-SMC3 antibodies (Bethyl Laboratories A300-055A)

SMC3 – polyclonal rabbit anti-SMC3 antibodies obtained from Jan-Michael Peters, described for immunoprecipitation and ChIP in [55] and [3].

MAU2 – polyclonal rabbit anti-MAU2(Scc4), described in [38].

RNA Pol II – polyclonal rabbit antibody (N-20) (Santa Cruz sc-899)

Tubulin – mouse anti-tubulin (Sigma)

Control IgG – rabbit whole serum

Rad21 – polyclonal rabbit anti-RAD21 (Jan-Michael Peters), described in [29]

### Cell culture

HeLa cells were cultured in DMEM supplemented with 0.2 mM L-glutamine, 100 units/ml penicillin, 100 mg/ml streptomycin and 10% FCS.

HB2 cells (1-7HB2, a clonal derivative of the human mammary luminal epithelial cell line MTSV1-7, [25]) were cultured in DMEM supplemented with 0.2 mM L-glutamine, 100 units/ml penicillin, 100 mg/ml streptomycin, 10% FCS, 5 µg/ml hydroxycortisone and 10 µg/ml human insulin.

Lymphoblastoid cell lines derived from controls and Cornelia de Lange syndrome patients (Fig. 5B) were obtained from Ian Krantz (The Children's Hospital of Philadelphia, Philadelphia, Pennsylvania, United States of America) and cultured in RPMI medium supplemented with 0.2 mM L-glutamine, 100 units per ml penicillin, 100 mg per ml streptomycin, 20% FCS.

SMC-LAP and Lamin-LAP Hela cells were were cultured in DMEM supplemented with 0.2 mM L-glutamine, 100 units/ml penicillin, 100 mg/ml streptomycin and 10% FCS and 0.2 mg/ml G418.

### RNAi depletion

The following siRNA oligos purchased form AMBION were used to deplete the respective proteins for ChIP-analysis and analysis of transcription

GL2

sense CGUACGCGGAAUACUUCGAtt

antisense UCGAAGUAUUCCGCGUACGtt

NIPBL

sense GCAUCGGUAUCAAGUCCCAtt

antisense UGGGACUUGAUACCGAUGCtt

MAU2

sense GCAUCGGUAUCAAGUCCCAtt

antisense UGGGACUUGAUACCGAUGCtt

SMC3

sense AUCGAUAAAGAGGAAGUUUtt

antisense AAACUUCCUCUUUAUCGAUtg

The following hairpin siRNA constructs in the pLKO.1–puro vector were obtained from the TRC Mission human library (Sigma) and were used to deplete NIPBL demonstrate the specificity of the NIPBL antibodies:

Control (clone SHC002) non-targeting sequence

NIPBL (clone TRCN0000129033) targeting sequence GCAGAGACAGAAGATGATGAA

The transfection of the siRNA oligos was performed with Lipofectamine RNAiMAX (Invitrogen) according to the manufacturer's instructions. The transfection of the hairpin siRNA constructs was performed with Lipofectamine 2000 (Invitrogen) according to the manufacturer's instructions. Cells were harvested 48 hours after transfection.

### Immunofluorescence staining

HeLa cells were grown on 18-mm cover slips and fixed with 4% PFA. After permeabelization with TX100 and blocking with 3% BSA the slides were stained with the respective antibodies.

Images were taken on a Leica DMRBE microscope equipped with a Hamatsu CCD (C4880) camera with a 100× objective. Images were processed with Image J, the colouring; overlay of the images was done with Adobe Photoshop.

### Cell cycle analysis

Cells were fixed with methanol and after RNAse treatment the DNA was stained with propidium iodine. The cells were analyzed with a BD FACS Aria Cell sorter and FlowJo software.

### Primers

See Table S6.

### Immunoprecipitation

To prepare nuclear extracts from HeLa cells the cells were first lysed by gentle resuspension in hypotonic buffer (20 mM Hepes-KOH pH 8.0, 5 mM KCl, 1.5 mM MgCl2, 0.1 mM DTT). Nuclei were collected by centrifugation and extracted for 30 min on ice with extraction buffer (15 mM Tris-HCl pH 7.5, 1 mM EDTA, 0.4 M NaCl, 10% sucrose, 0.01%TX-100, 1 mM DTT and 1 tablet Complete (Roche) per 50 ml buffer). Debris were removed by centrifugation (14000 rpm, 30 min).

The nuclear extract was diluted to 50% with IP buffer (20 mM Tris-HCl pH 7.5, 100 mM NaCl, 5 mM MgCl2, 0.2% NP40, 1 mM NaF, 0.5 mM DTT) and incubated for 1 h at 4°C with the respective antibodies. Affi-Prep Protein A support beads (BioRad) were added and incubated 1 h at 4°C. The beads were washed 3 times with IP buffer and eluted by boiling with SDS-page loading buffer. Western blots were analyzed with ECL+ reagent and Alliance imaging system.

### Chromatin immunoprecipitation

Chromatin immunoprecipitation was performed as described before [3]. In brief, cells at 70–80% confluence were crosslinked with 1% formaldehyde for 10 min and quenched with 125 mM glycine. After washing with PBS cells were resuspended in lysis buffer (50 mM Tris-HCl pH 8.0, 1% SDS, 10 mM EDTA, 1 mM PMSF and Complete protease inhibitor (Roche)) and chromatin was sonicated (Diagenode Bioruptor) to around 500 bp DNA fragments. Debris were removed by centrifugation, the lysate diluted 1∶4 with IP dilution buffer (20 mM Tris-HCl pH 8.0, 0.15 M NaCl, 2 mM EDTA, 1% TX-100, protease inhibitors) and precleared with Affi-Prep Protein A support beads (BioRad).

The respective antibodies were incubated with the lysate for 14 h at 4°C, followed by 2 h incubation at 4°C with blocked protein A Affiprep beads (Bio-Rad) (blocking solution: 0.1 mg/ml BSA or 0.1 mg/ml fish skin gelatine). The beads were washed with washing buffer I (20 mM Tris-HCl pH 8.0, 0.15 M NaCl, 2 mM EDTA, 1% TX-100, 0.1% SDS, 1 mM PMSF), washing buffer II (20 mM Tris-HCl pH 8.0, 0.5 M NaCl, 2 mM EDTA, 1% TX-100, 0.1% SDS, 1 mM PMSF), washing buffer III (10 mM Tris-HCl pH 8.0, 0.25 M LiCl, 1 mM EDTA, 0.5% NP-40, 0.5% sodium desoxycholate) and TE-buffer (10 mM Tris-HCl pH 8.0, 1 mM EDTA). The beads were eluted twice (25 mM Tris-HCl pH 7.5, 5 mM EDTA, 0.5% SDS) for 20 min at 65°C. The eluates were treated with proteinase K and RNase for 1 h at 37°C and decrosslinked 65°C over night. The samples were further purified by phenol-chloroform extraction and ethanol-precipitated. The pellet was dissolved in 50 µl TE buffer.

This protocol was used to perform ChIP-qPCR or ChIP-sequencing for CTCF, SMC3, NIPBL and RNA polymerase II. For SMC1A a milder ChIP protocol from Duncan Odom's group was used [26].

For NIPBL ChIP sequencing HB2 cells were synchronized in G1 phase by double thymidine block as described [8] (Suppl. Fig. S3). All other preparations were done from unsynchronized cells.

For NIPBL ChIP after depletion of NIPBL or control by RNAi the cells were synchronized in G1 phase by double thymidine block, starting 6 hours after transfection of the siRNA oligos. Details of the thymidine block to obtain HeLa cells in G1 phase are described [3].

Samples were either submitted for genomic sequencing or analyzed by qPCR using Platinium taq (Invitrogen) according to the manufacturer's instructions as described [3]. ChIP-qPCR experiments at least three times and one representative example is shown (SD was determined from qPCR replicates).

### ChIP sequencing and peak detection

The ChIP DNA library was prepared according to the Illumina protocol (www.illumina.com). Briefly, 10 ng of ChIPped DNA was end-repaired, ligated to adapters, size selected on gel (200±25 bp range) and PCR amplified using Phusion polymerase as follow: 30 sec at 98°C, 18 cycles of (10 sec at 98°C, 30 sec at 65°C, 30 sec at 72°C), 5 min at 72°C final extension. Cluster generation was performed using the Illumina Cluster Reagents preparation. The libraries for NIPBL, CTCF, SMC3, RNA PolII and the respective controls generated from HB2 cells were sequenced on the Illumina Genome Analyzer II, the SMC1A ChIP samples from HB2 cells, the NIPBL ChIP samples from LCLs and the respective controls were sequenced with the Illumina HiSeq2000 system. Read lengths of 36 bases were obtained. Images were recorded and analyzed by the Illumina Genome Analyzer Pipeline (GAP 1.6.0. and 1.7.0.). The resulting sequences were mapped against Human_UCSChg18 using the Bowtie [56] alignment software, with the following parameters: bowtie -m 1 -S -k 1 –n 1. Unique reads were selected for further analysis.

Peak calling for the ChIP sequencing data was performed with SWEMBL (URL: http://www.ebi.ac.uk/~swilder/SWEMBL/) as described [37] with the respective parameters described in Table S1.

Co-localization read density profiles were done by extending a region around each peak summit by +/−200 bp. Regions from each data set were chosen in succession as viewpoints, and the position of 5′ends of the reads in corresponding regions in all data sets was plotted. The profiles were ordered by the significance score determined by the Swembl peak caller.

#### Peak annotation

Complete Ensembl hg18 gene dataset was downloaded on 13.04.2011. The genome was separated into 4 regions: promoter (+/−1 kb from the TSS), upstream (−5000 from the TSS), downstream (+5000 from the gene end) and gene body. A region of +/−150 bp was extended around each peak and overlapped with the genomic annotation.

Peaks were designated into one category based on the following order of preference: promoter→gene body→upstream→downstream.

### Repeat analyses

To investigate the repeat enrichment pattern, we used both uniquely- and multiply-aligned reads. Multiply-aligned reads were divided equally amongst all locations (N-times matched reads were weighted as 1/N reads). The reads which were aligned to reference genome more than 10 times were discarded. We applied RPKM measure (reads per kilobase per million reads) which was utilized for RNA-seq analyses [56], but we used “per 10 million reads” instead of “per million reads”. We counted the reads which were aligned to each repeat class and normalized the counts against the total number of aligned reads (whole-genome) and the total length of each repeat class.

### RNA sequencing

HB2 cells were enriched in G1 phase by double thymidine block as described [8]. The RNA was isolated using TRI reagent (Sigma) according to the supplier's protocol. Two microgram of total RNA was converted into a library of template molecules suitable for sequencing according to the Illumina mRNA Sequencing sample prep protocol. Briefly, polyA containing mRNA molecules were purified using poly-T oligo attached magnetic beads. Following purification, the mRNA is fragmented into ∼200 bp fragments using divalent cations under elevated temperature. The cleaved RNA fragments are copied into first strand cDNA using reverse transcriptase and random primers. This is followed by second strand synthesis using DNA polymerase I and RNaseH treatment. These cDNA fragments are end repaired, a single A base is added and Illumina adaptors are ligated. The products are purified and size selected on gel and enriched by PCR. The PCR products are purified by Qiaquick PCR purification and used for cluster generation according to the Illumina cluster generation protocols (www.illumina.com). The sample was sequenced for 36 bp and raw data was processed using Narwhal [57].

### RNA sequencing analysis

RNA Seq reads were mapped to the Human UCSChg18 genome with Bowtie using the same parameters as for the ChIP seq analysis. The coverage vector was calculated from unique reads and the expression value was determined for each gene by taking the RPKM [58] of the most highly expressed isoform (the sum of coverage over exons was used as the numerator of the equation). All genes with RPKM>0.6 were designated as expressed.

### Motif analysis

Motif analysis was performed with the tools MEME and MEME-ChIP [33]. Residues +/−50 bp of NIPBL binding site peaks were retrieved and submitted to MEME-ChIP using standard parameters.

To analyse whether the presence of the NFYA motif at NIPBL sites is due to the presence of CpG islands or is a genuine property of NIPBL binding we selected NIPBL binding sites close to only one CpG island promoter (692 sites) and selected the same number of CpG island promoters at random. The presence of the NFYA motif was detected and the counts statistically analyzed using a Fischer-test.

### Identification of colocalizing transcription factors

We obtained from ENCODE [32] ChIP-sequencing data tracks for transcription factors generated from GM12878 cells and deposited by the Myers lab (HudsonAlpha Institute for Biotechnology) and the Snyder lab (Yale University). When called peaks were available they were used, else replicates were pooled and peak calling performed with MACS [58]. Peaks were sorted for intensity and for the 10000, 5000 and 1000 (in case of NIPBL) strongest peaks heatmaps were generated centred on NIPBL binding sites conserved in the different lymphoblastoid cell lines and also centred on the peaks of the respective transcription factors. Overlapping patterns were selected by visual inspection of the maps.

#### Myers lab (Haib)

ATF2, ATF3, BATF, BCL1, BCL3, BCLAF, BHLH, BRCA1, CFOS, CHD2, CTCF, EBF1, EGR1, ELF1, ETS1, FOXM1, GABP, GCN5, IRF3, IRF4, JUND, MAX, MEF2, MTA3, MXI1, NFATC1, NFE2, NFIC, NFYA, NFYB, NRF1, NRSF, p300, PAX5, PBX3, PML, Pol2, Pol3, POU2, PU.1, RAD21, RFX, RUNX3, RXLCH, RXRA, SIX5, SMC3, SP1, SPT, SRF, STAT1, STAT3, STAT5, TBP, TCF1, TCF3, TR4, USF1, USF2, WHIP, YY1, ZBTB3, ZEB1, ZNF143, ZNF274, ZZZ3

#### Snyder lab (SYDH)

BHLH, BRCA1, CFOS, CHD2, CTCF, EBF1, GCN5, IRF3, JUND, MAX, MXI1, NFE2, NFYA, NFYB, NRF1, p300, RAD21, RFX, SMC3, SPT, STAT1, STAT3, TBP, TR4, USF2, WHIP, YY1, ZNF143, ZNF274, ZZZ3

### Transcript analysis

HB2 cells were transfected with the respective siRNA oligos using Lipofectamine 2000 and were harvested after 48 hours. The RNA was prepared using TRI reagent (Sigma). Remaining DNA was removed by DNAse treatment and cDNA synthesis was performed with Superscript reverse Transcriptase (Invitrogen) using oligo-dT primers. The qPCR analysis was performed as described [3].

### Ethics statement

This study was conducted according to the principles expressed in the Declaration of Helsinki. The study was approved by the Institutional Review Board of The Children's Hospital of Philadelphia. All patients provided written informed consent for the collection of samples and subsequent analysis.

## Supporting Information

Figure S1 Cohesin loading occurs after nuclear envelop reformation. (A) To test and visualize the specificity of the antibodies used for the immunostaining experiments HeLa cells were treated with the respective siRNA for NIPBL, RAD21 and CTCF and then seeded on cover slips in a mix with control siRNA-treated cells to visualize the effect of the RNAi depletion next to the control cells. The slides were stained with anti-NIPBL #4, anti-RAD21 and anti-CTCF and for each secondary antibody a control slide without primary antibody was included. The undepleted cells are marked with white arrows in the antibody-stained slides. (B, C) LaminA-LAP expressing HeLa cells (EGFP, green) were stained with antibodies against (panel B) SA2/STAG2 (red) and (panel C) NIPBL (red). Images were taken from interphase cells (I) and different stages during the exit from mitosis (anaphase (A), late anaphase (LA), telophase (T) and early G1 phase (EG1). In panel B the cohesin signal can only be observed overlapping with chromatin when a nuclear envelop is visible (white arrows in telophase and early G1 phase cells). In contrast the NIPBL signal in panel C appears on chromatin already before a nuclear envelop is visible (white arrows in late anaphase cells).
(PDF)

Figure S2 Characterization of NIPBL antibodies. We first characterized different antibodies raised against NIPBL, a 320 kDa protein that is difficult to detect by immunoblotting and immunofluorescense staining. For detection by western blotting we used two rat monoclonal antibodies against the two major isoforms of NIPBL, Isoform A (NP_597677, NIPBL#3) and Isoform B (NP_056199, NIPBL#4). The isoforms are splice variants of the last exon, residues 1–2683 are identical but isoform A contains 121 and isoform B 14 unique C-terminal residues. (A) Western blot showing that the band recognized by NIPBL#4 can be depleted by NIPBL-specific siRNA in unsynchronized HeLa cells while it remains well visible in two control siRNA transfections. (B) Immunoprecipitations with the rabbit anti-NIPBL antibodies NIPBL#1 and NIPBL#6 antibodies and anti-SMC3 antibodies were performed from nuclear extract of G1-phase enriched HeLa cells. Two identical western blots were generated which were probed with rat monoclonal antibodies against the two isoforms of NIPBL (NIPBL#3 for isoform A and NIPBL#4 for isoform B) and one re-probed with anti-SMC1 (rabbit) after quenching of the rat antibody signal. Both isoform-specific antibodies detected one major (>250 kDa) and minor NIPBL bands in the G1-phase nuclear extracts (input lane). Multiple bands for NIPBL could occur due to posttranslational modifications of NIPBL. Significant difference between NIPBL#1 and #6 are visible in the immunoprecipitates. NIPBL#1, used by us for ChIP-seq, immunoprecipitates all bands, while NIPBL#6, used by Kagey et al. [13] for ChIP-seq from mouse ES cells, precipitates only the lower bands. We concluded that the NIPBL#1 antibody recognizes a wider spectrum of NIPBL (posttranslationally modified) forms. Interestingly, the antibody against the cohesin subunit (SMC3) did not precipitate any of the NIPBL isoforms (Fig. 1C), consistent with previous observations of very weak interactions between NIPBL and cohesin [38].
(PDF)

Figure S3 Determination of cell cycle stages by FACS analysis. (A) HB2 cells growing logarithmically or enriched in G1 phase for NIPBL ChIP were fixed with methanol, stained for the DNA content with propidium iodine and analyzed by FACS. (B) HB2 cells treated with different siRNA's were enriched in G2 phase. Cells were fixed with methanol, stained for the DNA content with propidium iodine and analyzed by FACS.
(PDF)

Figure S4 Specificity of the NIPBL antibody used for ChIP-sequencing. (A) Genomic binding of NIPBL in a selected region on chromosome 19 in comparison between HB2 cells and HeLa cells. Both cell lines were enriched in G1 phase for the ChIP-sequencing experiment. The position of the peaks is similar between HB2 and HeLa cells, although the enrichment in HeLa was much weaker. As controls the sequencing data from the respective input materials are shown. (B) Western blot showing the depletion of NIPBL in HeLa cells. Since MAU2 is also destabilized when NIPBL is depleted it can be used as marker for NIPBL depletion [38], which is rather difficult to blot. The band indicated with * is an unspecific signal of the MAU2 antibodies and can be used as loading control. (C) NIPBL and control ChIP was performed from HeLa cells treated with NIPBL and control siRNA. QPCR analysis with primers specific for several NIPBL binding sites identified in HB2 cells shows that NIPBL RNAi dramatically reduces the NIPBL ChIP signal. The experiment was performed three times and one representative example is shown. (D) HeLa cells were treated with control and NIPBL RNAi and stained with different antibodies against NIPBL (green – NIPBL#1, rabbit polyclonal; red - NIPBL#3, rat monoclonal) and with DAPI to visualize DNA. Both antibodies show similar reduction of the signal after NIPBL RNAi, indicating that both recognize the same target protein. The images we selected to show also cells not targeted by the siRNA to visualize the efficiency of the depletion.
(PDF)

Figure S5 Localization of NIPBL to repeats and comparison of the NIPBL#1 and NIPBL#6 antibodies in mouse ES cells. (A) ChIP/q-PCR validation of NIPBL-binding sites on repetitive regions in HB2 cells. The experiment was performed in duplicate and both samples are shown. Five primers for LSU repeats (LSU) and three for SSU repeats (SSU) and one negative control region (*AMY*) were analysed. (B, C) To compare both anti-NIPBL antibodies we performed ChIP from mouse ES cells using the protocol by Kagey et al. (upper panel) (B) and our protocol (C). We tested several NIPBL sites at promoters that were identified by Kagey et al. (*Nanog*, *Lefty1*, *Oct4*) and by our study (*Tiam1*, *Ankhd1*, *Sp1*; initially identified in HB2 cells but found to be it conserved in mouse ES cells). The left panel in (C) shows the full plot and the right panel a zoom-in on the %IP of input values up to 0.05%IP of input to visualize the ChIP performance at the “minor” low affinity binding sites identified by Kagey et al.(mean n = 2, ± s.d.). (D) Immunostaining of mouse ES cells derived from *Nipbl +/−* embryos for ES cell markers. (E) ChIP with NIPBL#1 (left panel) and #6 antibodies (right panel) from control (IB10) and *NIPBL +/−* mouse ES cells (S. Goldberg, F. Grosveld unpublished data) shows reduced Nipbl binding levels in Nipbl *NIPBL +/−* cells detected by both antibodies. (mean n = 2, ± s.d.). (F) To compare the ChIP efficiency of the NIPBL#6 antibodies with NIPBL#1 in human cells we performed ChIP with NIPBL #6 from HB2 cells (right panel) and compared it with the ChIP example also showed in figure 2 E (left panel). (mean n = 2, ± s.d.)
(PDF)

Figure S6 NIPBL-binding is reduced in LCL cells derived from CdLS patients. NIPBL (NIPBL#1) and negative control ChIP (IgG) was performed from lymphoblastoid cells derived from CdLS patients and age-matched controls and analyzed by qPCR with primers specific for seven NIPBL binding sites, one cohesin binding site and a negative control site (*AMY*). The sites analysed are indicated above the graph. The enrichment compared to the control IgG ChIP was calculated. The experiment was performed more than three times and a representative example is shown.
(PDF)

Table S1 Parameters used for peak calling with SWEMBL.
(PDF)

Table S2 Peaks identified with SWEMBL in the different datasets.
(PDF)

Table S3 Position of the binding sites identified for the different proteins relative to genes. Around each gene four regions were considered to cluster the binding sites; upstream – −5000 to −1000 bp from the transcription start site (TSS); promoter – −1000 to +1000 bp from TSS; gene body – +1000 bp from TSS until the end of the coding sequence; downstream – from the end of the coding sequence to +5000 bp. Gene activity was scored based on RPKM and genes with RPKM>0.6 were considered as expressed.
(PDF)

Table S4 Classification of promoters and expression status of genes bound by NIPBL, cohesin (SMC1A and SMC3), RNA Pol II and CTCF. The region −1000 to + 1000 bp from TSS was considered as promoter region.
(PDF)

Table S5 Functional annotation of genes with NIPBL binding sites in HB2 cells by IPA analysis.
(PDF)

Table S6 Primers used for ChIP/qPCR and RT-PCR/qPCR.
(PDF)

Table S7 Genes with NIPBL binding sites in a patient-derived lymphoblastoid cells (PT1) found to be differentially expressed in CdLS patients with FDR<0.05 by Liu et al., 2009 (18).
(PDF)

Table S8 NIPBL ChIP signals in HB2 cells on different repeat classes. RPKM measure (reads per kilobase per 10 million reads) was calculated similar to the RNA-seq analyses (56) and an enrichment compared to the input material (control) was calculated.
(PDF)
