# Supplementary figures and images for: A Cohesin-Independent Role for NIPBL at Promoters Provides Insights in CdLS
Source: PLoS Genet. 2014 Feb 13;10(2):e1004153. doi: 10.1371/journal.pgen.1004153 (PMC3923681; doi:10.1371/journal.pgen.1004153)

A

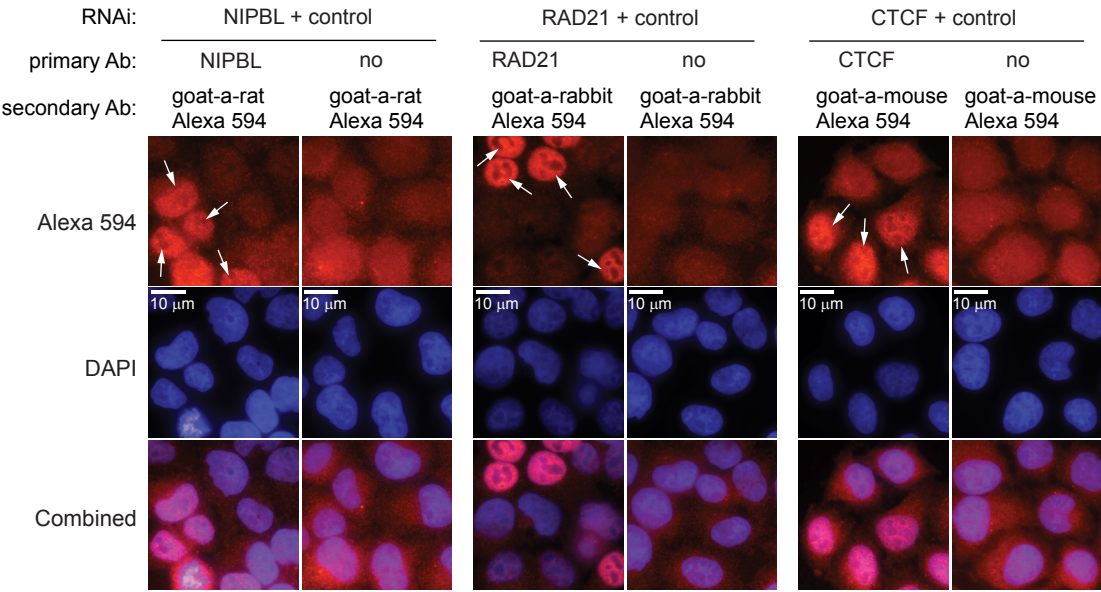

B

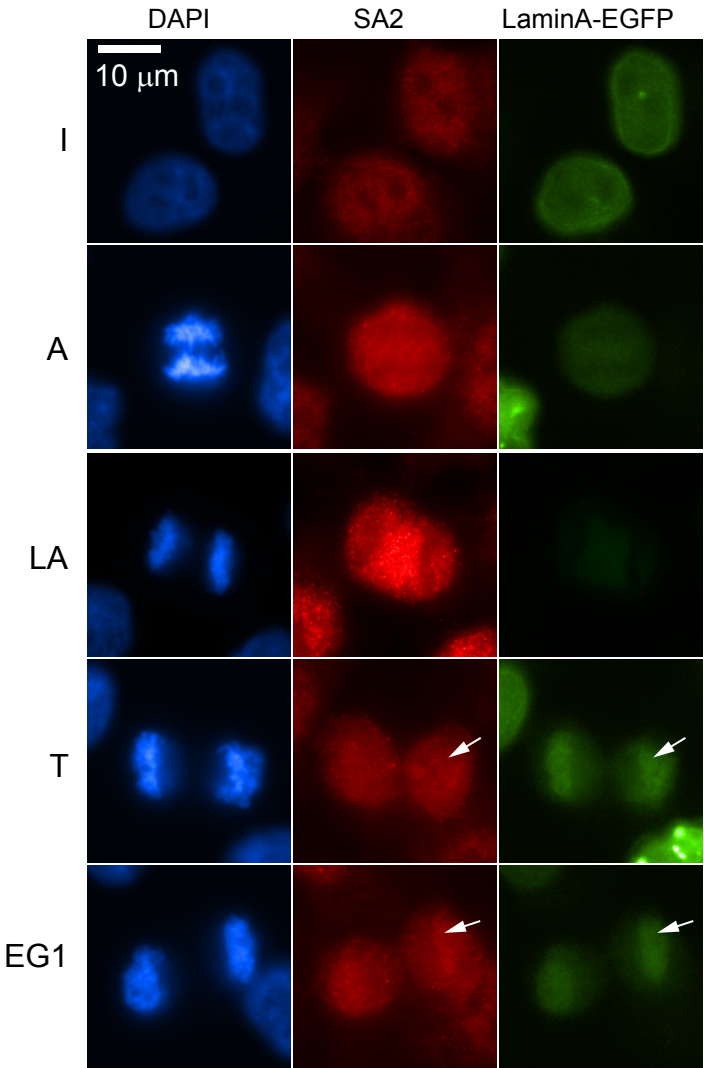

C

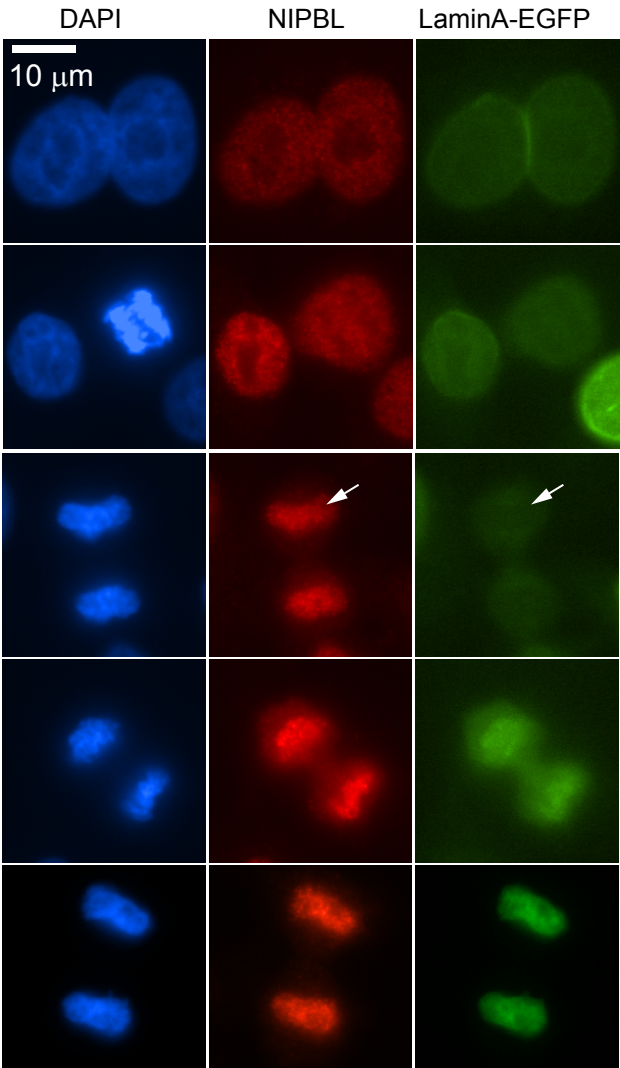

I     interphase  
A     anaphase  
LA    late anaphase  
T     telophase  
EG1   early G1 phase

Supplement: Figure S1 — Cohesin loading occurs after nuclear envelop reformation. (A) To test and visualize the specificity of the antibodies used for the immunostaining experiments HeLa cells were treated with the respective siRNA for NIPBL, RAD21 and CTCF and then seeded on cover slips in a mix with control siRNA-treated cells to visualize the effect of the RNAi depletion next to the control cells. The slides were stained with anti-NIPBL #4, anti-RAD21 and anti-CTCF and for each secondary antibody a control slide without primary antibody was included. The undepleted cells are marked with white arrows in the antibody-stained slides. (B, C) LaminA-LAP expressing HeLa cells (EGFP, green) were stained with antibodies against (panel B) SA2/STAG2 (red) and (panel C) NIPBL (red). Images were taken from interphase cells (I) and different stages during the exit from mitosis (anaphase (A), late anaphase (LA), telophase (T) and early G1 phase (EG1). In panel B the cohesin signal can only be observed overlapping with chromatin when a nuclear envelop is visible (white arrows in telophase and early G1 phase cells). In contrast the NIPBL signal in panel C appears on chromatin already before a nuclear envelop is visible (white arrows in late anaphase cells). (PDF) [file pgen.1004153.s001.pdf]

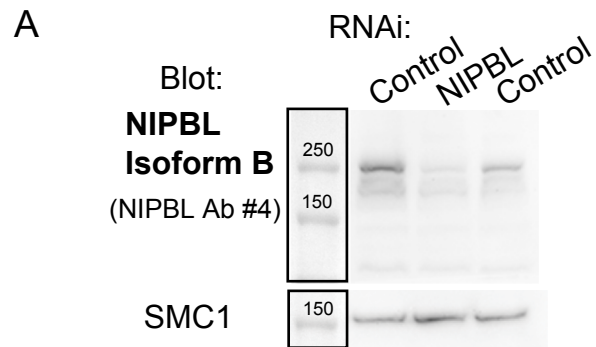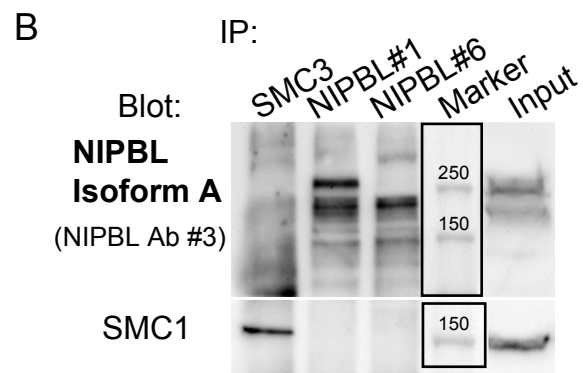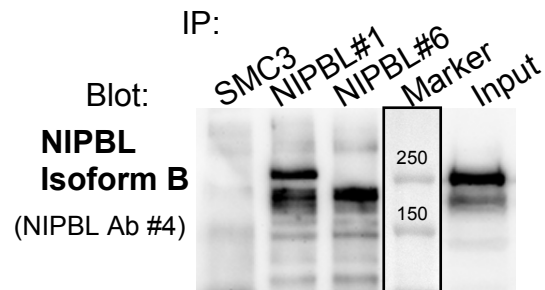

Supplement: Figure S2 — Characterization of NIPBL antibodies. We first characterized different antibodies raised against NIPBL, a 320 kDa protein that is difficult to detect by immunoblotting and immunofluorescense staining. For detection by western blotting we used two rat monoclonal antibodies against the two major isoforms of NIPBL, Isoform A (NP_597677, NIPBL#3) and Isoform B (NP_056199, NIPBL#4). The isoforms are splice variants of the last exon, residues 1–2683 are identical but isoform A contains 121 and isoform B 14 unique C-terminal residues. (A) Western blot showing that the band recognized by NIPBL#4 can be depleted by NIPBL-specific siRNA in unsynchronized HeLa cells while it remains well visible in two control siRNA transfections. (B) Immunoprecipitations with the rabbit anti-NIPBL antibodies NIPBL#1 and NIPBL#6 antibodies and anti-SMC3 antibodies were performed from nuclear extract of G1-phase enriched HeLa cells. Two identical western blots were generated which were probed with rat monoclonal antibodies against the two isoforms of NIPBL (NIPBL#3 for isoform A and NIPBL#4 for isoform B) and one re-probed with anti-SMC1 (rabbit) after quenching of the rat antibody signal. Both isoform-specific antibodies detected one major (>250 kDa) and minor NIPBL bands in the G1-phase nuclear extracts (input lane). Multiple bands for NIPBL could occur due to posttranslational modifications of NIPBL. Significant difference between NIPBL#1 and #6 are visible in the immunoprecipitates. NIPBL#1, used by us for ChIP-seq, immunoprecipitates all bands, while NIPBL#6, used by Kagey et al. [13] for ChIP-seq from mouse ES cells, precipitates only the lower bands. We concluded that the NIPBL#1 antibody recognizes a wider spectrum of NIPBL (posttranslationally modified) forms. Interestingly, the antibody against the cohesin subunit (SMC3) did not precipitate any of the NIPBL isoforms (Fig. 1C), consistent with previous observations of very weak interactions between NIPBL and cohesin [38]. (PDF) [file pgen.1004153.s002.pdf]

**A**

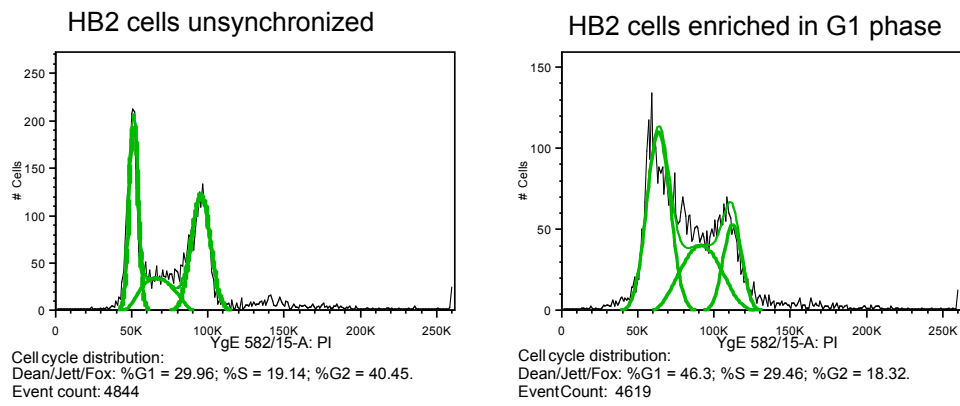

**B**

HB2 cells treated with different siRNA and enriched in G2 phase by double thymidine block

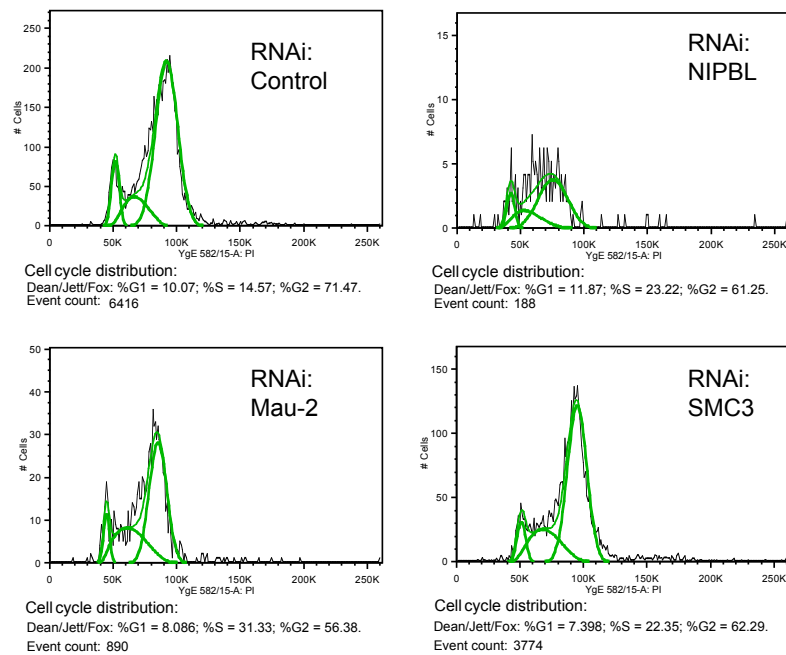

Supplement: Figure S3 — Determination of cell cycle stages by FACS analysis. (A) HB2 cells growing logarithmically or enriched in G1 phase for NIPBL ChIP were fixed with methanol, stained for the DNA content with propidium iodine and analyzed by FACS. (B) HB2 cells treated with different siRNA's were enriched in G2 phase. Cells were fixed with methanol, stained for the DNA content with propidium iodine and analyzed by FACS. (PDF) [file pgen.1004153.s003.pdf]

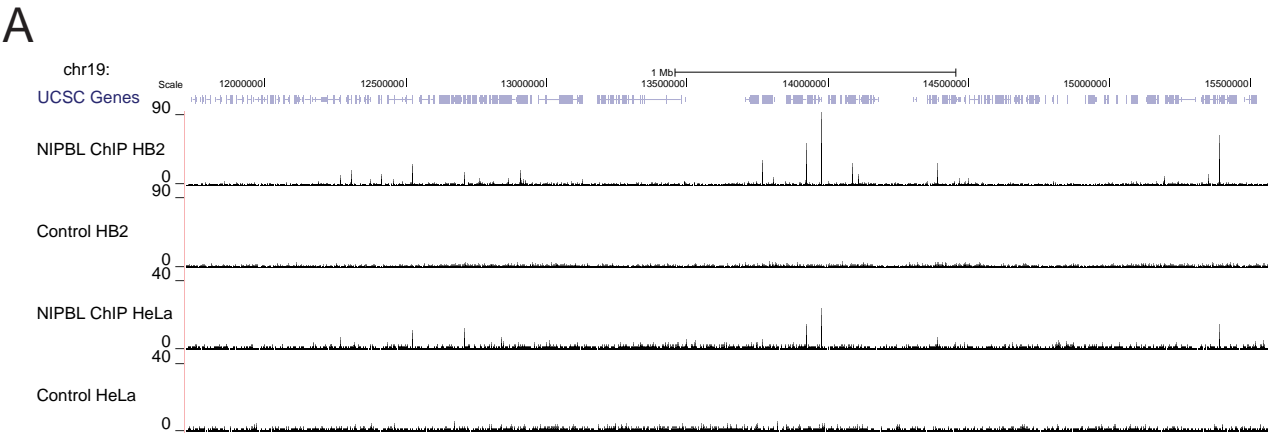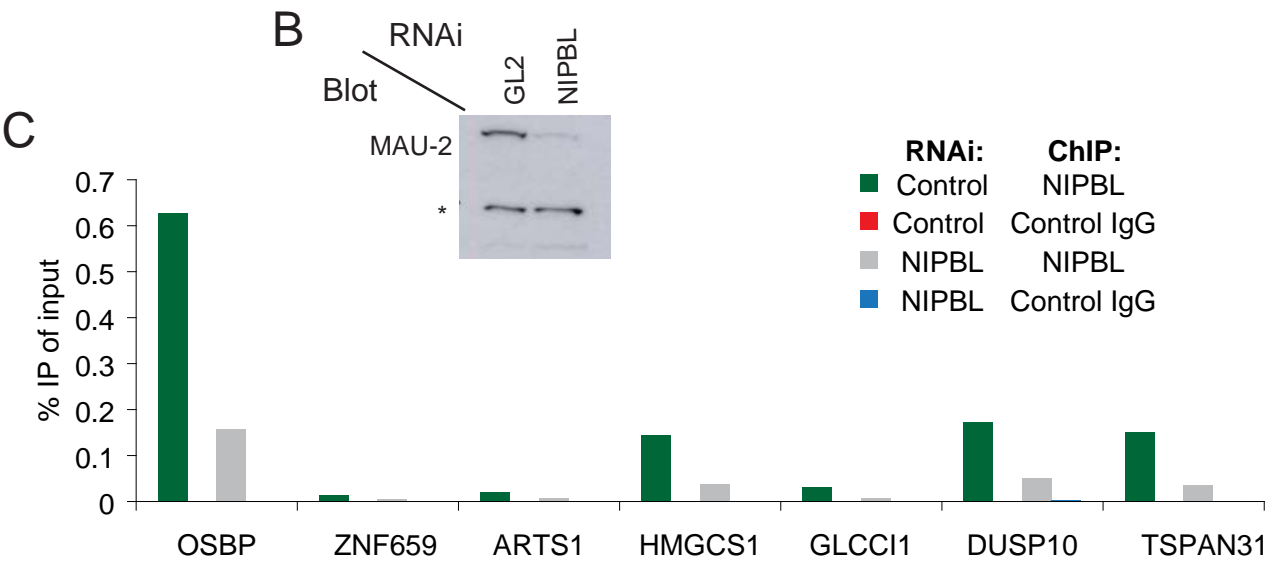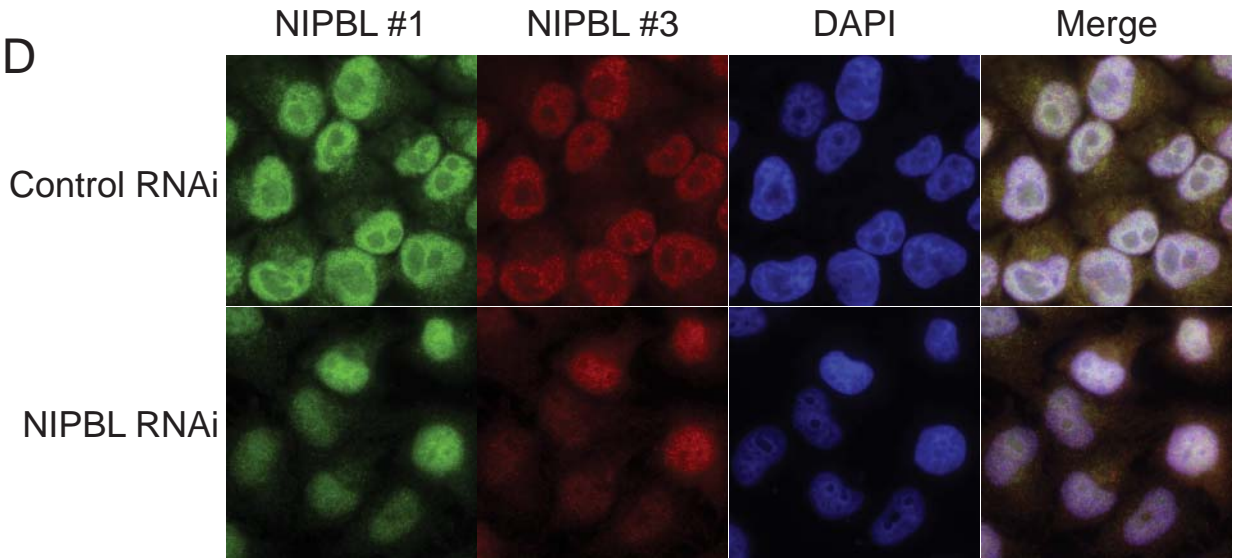

Supplement: Figure S4 — Specificity of the NIPBL antibody used for ChIP-sequencing. (A) Genomic binding of NIPBL in a selected region on chromosome 19 in comparison between HB2 cells and HeLa cells. Both cell lines were enriched in G1 phase for the ChIP-sequencing experiment. The position of the peaks is similar between HB2 and HeLa cells, although the enrichment in HeLa was much weaker. As controls the sequencing data from the respective input materials are shown. (B) Western blot showing the depletion of NIPBL in HeLa cells. Since MAU2 is also destabilized when NIPBL is depleted it can be used as marker for NIPBL depletion [38], which is rather difficult to blot. The band indicated with * is an unspecific signal of the MAU2 antibodies and can be used as loading control. (C) NIPBL and control ChIP was performed from HeLa cells treated with NIPBL and control siRNA. QPCR analysis with primers specific for several NIPBL binding sites identified in HB2 cells shows that NIPBL RNAi dramatically reduces the NIPBL ChIP signal. The experiment was performed three times and one representative example is shown. (D) HeLa cells were treated with control and NIPBL RNAi and stained with different antibodies against NIPBL (green – NIPBL#1, rabbit polyclonal; red - NIPBL#3, rat monoclonal) and with DAPI to visualize DNA. Both antibodies show similar reduction of the signal after NIPBL RNAi, indicating that both recognize the same target protein. The images we selected to show also cells not targeted by the siRNA to visualize the efficiency of the depletion. (PDF) [file pgen.1004153.s004.pdf]

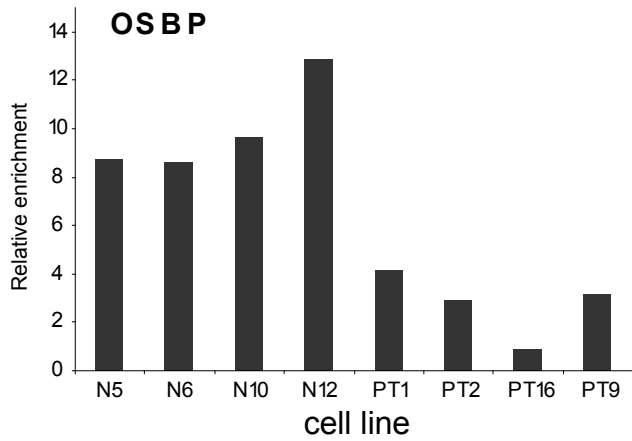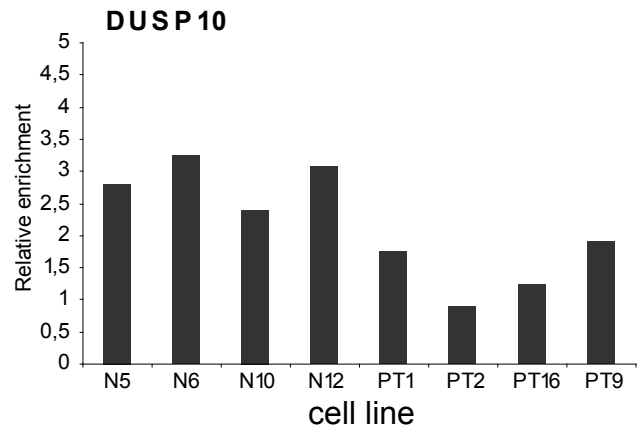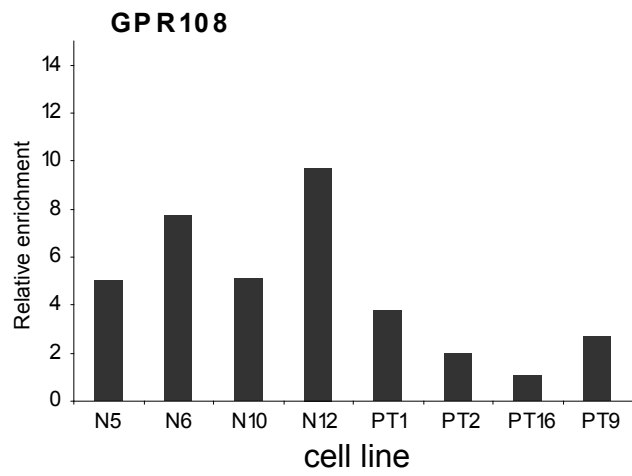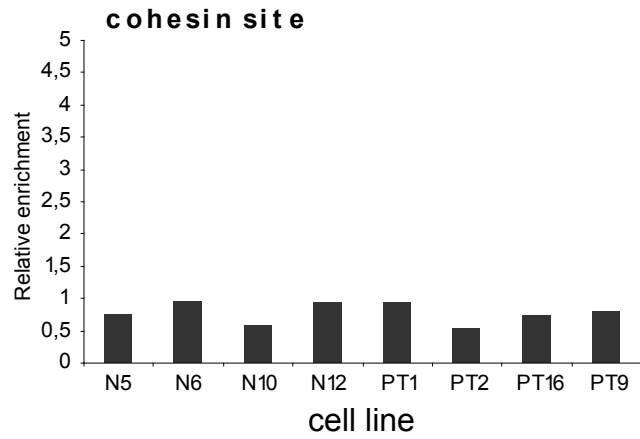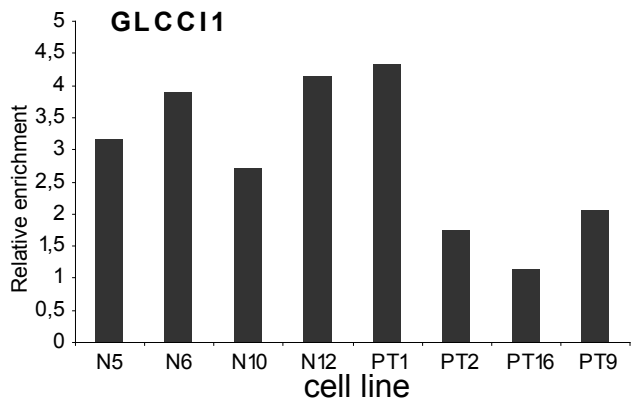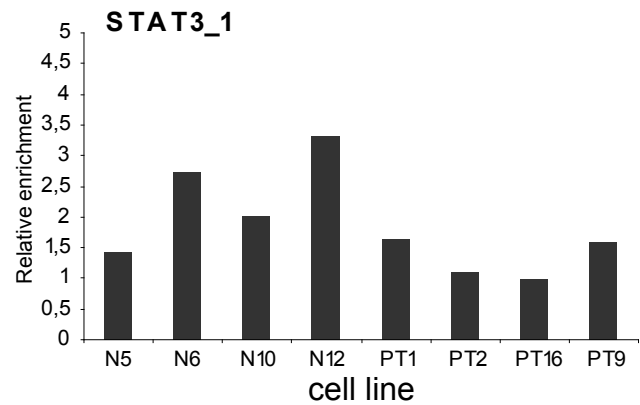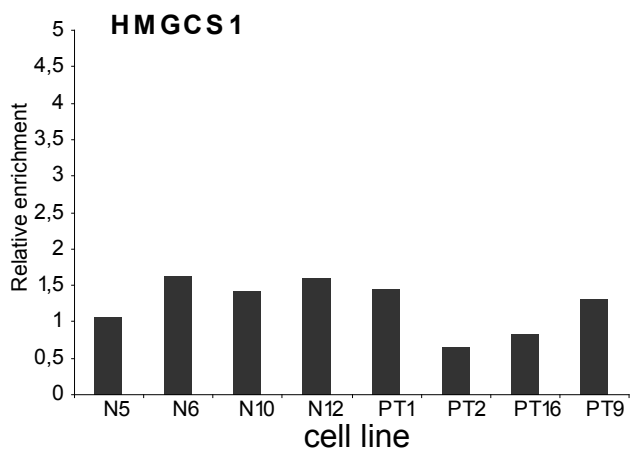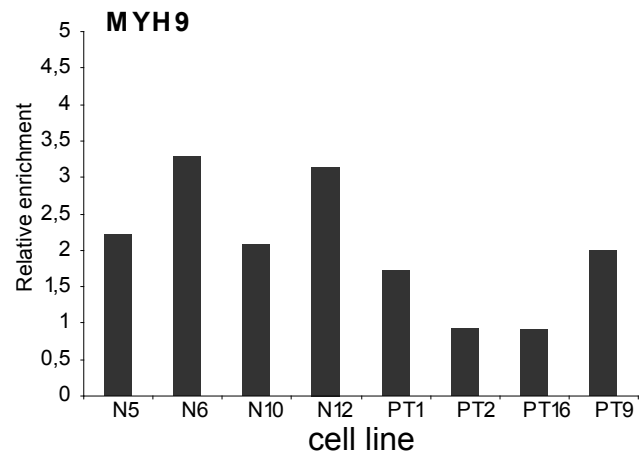

Supplement: Figure S6 — NIPBL-binding is reduced in LCL cells derived from CdLS patients. NIPBL (NIPBL#1) and negative control ChIP (IgG) was performed from lymphoblastoid cells derived from CdLS patients and age-matched controls and analyzed by qPCR with primers specific for seven NIPBL binding sites, one cohesin binding site and a negative control site (AMY). The sites analysed are indicated above the graph. The enrichment compared to the control IgG ChIP was calculated. The experiment was performed more than three times and a representative example is shown. (PDF) [file pgen.1004153.s006.pdf]
