# Supplementary material for: A Cohesin-Independent Role for NIPBL at Promoters Provides Insights in CdLS
Source: PLoS Genet. 2014 Feb 13;10(2):e1004153. doi: 10.1371/journal.pgen.1004153 (PMC3923681; doi:10.1371/journal.pgen.1004153)

Zuin et al., Figure S5

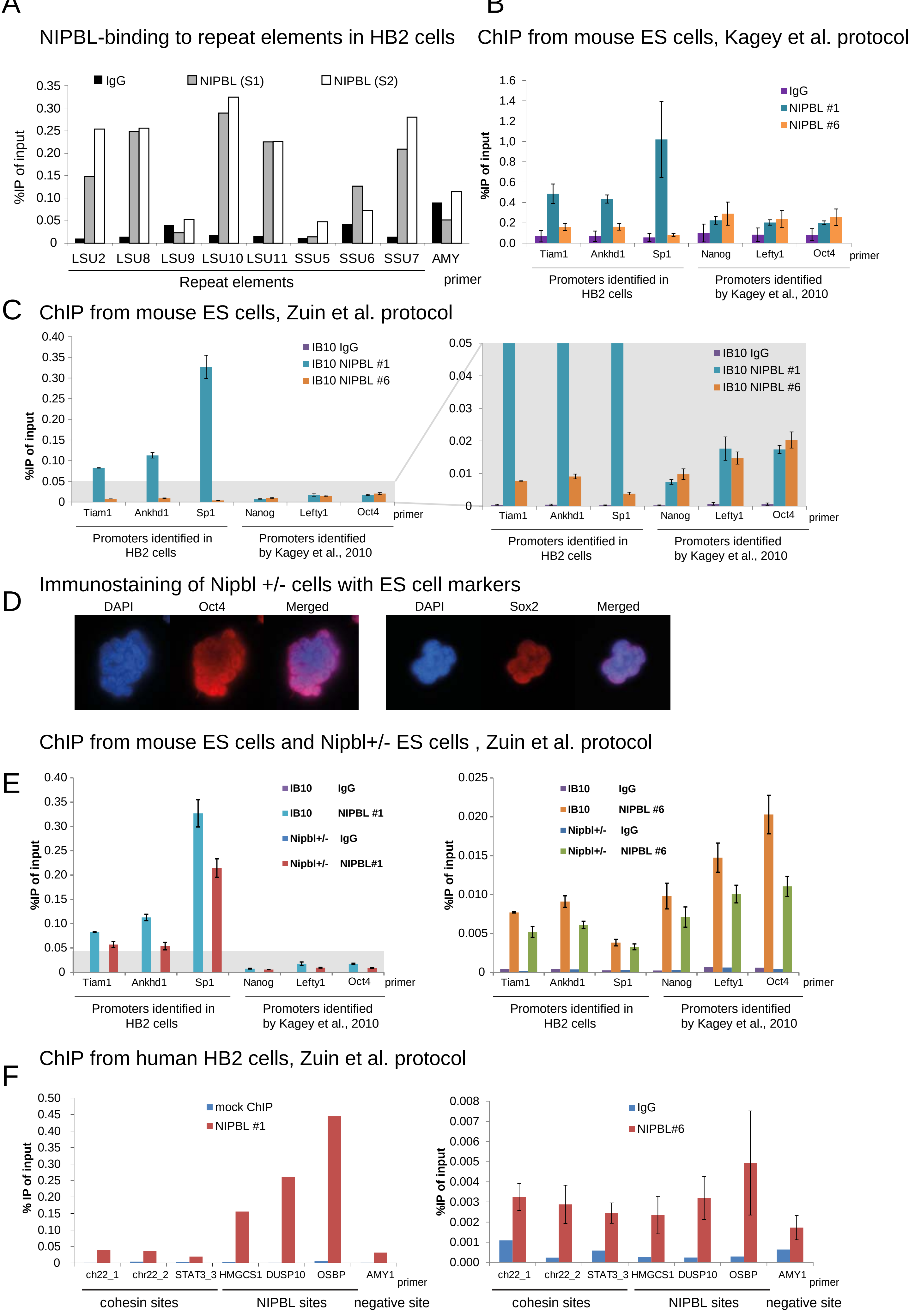

Supplement: Figure S5 — Localization of NIPBL to repeats and comparison of the NIPBL#1 and NIPBL#6 antibodies in mouse ES cells. (A) ChIP/q-PCR validation of NIPBL-binding sites on repetitive regions in HB2 cells. The experiment was performed in duplicate and both samples are shown. Five primers for LSU repeats (LSU) and three for SSU repeats (SSU) and one negative control region (AMY) were analysed. (B, C) To compare both anti-NIPBL antibodies we performed ChIP from mouse ES cells using the protocol by Kagey et al. (upper panel) (B) and our protocol (C). We tested several NIPBL sites at promoters that were identified by Kagey et al. (Nanog, Lefty1, Oct4) and by our study (Tiam1, Ankhd1, Sp1; initially identified in HB2 cells but found to be it conserved in mouse ES cells). The left panel in (C) shows the full plot and the right panel a zoom-in on the %IP of input values up to 0.05%IP of input to visualize the ChIP performance at the “minor” low affinity binding sites identified by Kagey et al.(mean n = 2, ± s.d.). (D) Immunostaining of mouse ES cells derived from Nipbl +/− embryos for ES cell markers. (E) ChIP with NIPBL#1 (left panel) and #6 antibodies (right panel) from control (IB10) and NIPBL +/− mouse ES cells (S. Goldberg, F. Grosveld unpublished data) shows reduced Nipbl binding levels in Nipbl NIPBL +/− cells detected by both antibodies. (mean n = 2, ± s.d.). (F) To compare the ChIP efficiency of the NIPBL#6 antibodies with NIPBL#1 in human cells we performed ChIP with NIPBL #6 from HB2 cells (right panel) and compared it with the ChIP example also showed in figure 2 E (left panel). (mean n = 2, ± s.d.) (PDF) [file pgen.1004153.s005.pdf]
