# Supplementary material for: A Cohesin-Independent Role for NIPBL at Promoters Provides Insights in CdLS
Source: PLoS Genet. 2014 Feb 13;10(2):e1004153. doi: 10.1371/journal.pgen.1004153 (PMC3923681; doi:10.1371/journal.pgen.1004153)
Supplement: Table S1 — Parameters used for peak calling with SWEMBL. (PDF) [file pgen.1004153.s007.pdf]

Zuin et al., Table S1

Parameters for peak calling with SWEMBL

	CTCF	RNA Pol II	SMC1A	SMC3	NIPBL
#Sequence length	36	36	36	36	36
#Fragment length	150	150	150	150	150
#Background	0.000000	0.000000	0.000000	0.000000	0.000000
#Position Background	0.010000	0.010000	0.010000	0.010000	0.010000
#Long Background	0.050000	0.050000	0.050000	0.050000	0.050000
#Threshold	5.000000	5.000000	5.000000	5.000000	5.000000
#Minimum count above bg	10	10	15	8	10
#Penalty increase	25	25	25	25	25
#Quality cutoff	0.000000	0.000000	0.000000	0.000000	0.000000
#Result cutoff	4.000000	4.000000	8.000000	4.000000	4.000000
#Penalty factor	1.000000	1.000000	1.000000	1.000000	1.000000
