# Supplementary material for: A Cohesin-Independent Role for NIPBL at Promoters Provides Insights in CdLS
Source: PLoS Genet. 2014 Feb 13;10(2):e1004153. doi: 10.1371/journal.pgen.1004153 (PMC3923681; doi:10.1371/journal.pgen.1004153)
Supplement: Table S2 — Peaks identified with SWEMBL in the different datasets. (PDF) [file pgen.1004153.s008.pdf]

Zuin et al., Table S2

A Peaks identified with SWEMBL in the different datasets

	CTCF	SMC1A	SMC3	RNA Pol II	NIPBL
Total number of peaks	35672	29448	22573	9897	1138
Number of peaks associated with genes*	21743	16501	13657	9148	1016
% of peaks associated with genes	61	56	61	92	89
Number of peaks associated with **Expressed** genes	14501	10775	9801	8802	953
% of peaks associated with **Expressed** genes	41	37	43	89	84

* as peaks were counted as associated with genes when they matched to one of the following groups:
upstream: -5000 to -1000 bp from transcription start site
promoter: -1000 to +1000 bp around transcription start site
gene body: +1000 bp from transcription start site until end of the coding sequence
downstream: end of the coding sequence + 5000 bp

B Overlap statistics (%)

		Percent overlap in a window of +/- 100 bp:				
**Total number of sites**		**CTCF**	**SMC1A**	**SMC3**	**NIPBL**	**SMC1A+SMC3**
**CTCF**	**35672**		51.4	38.3	0.3	59.3
**SMC1A**	**29448**	62.3		39.2	0.2	100.0
**SMC3**	**22573**	60.5	51.1		0.4	100.0
**NIPBL**	**1138**	9.8	4.3	8.4		10.4
**SMC1+SMC3**	**40465**	52.3	72.8	55.8	0.3	
