# Supplementary material for: A Cohesin-Independent Role for NIPBL at Promoters Provides Insights in CdLS
Source: PLoS Genet. 2014 Feb 13;10(2):e1004153. doi: 10.1371/journal.pgen.1004153 (PMC3923681; doi:10.1371/journal.pgen.1004153)
Supplement: Table S3 — Position of the binding sites identified for the different proteins relative to genes. Around each gene four regions were considered to cluster the binding sites; upstream – −5000 to −1000 bp from the transcription start site (TSS); promoter – −1000 to +1000 bp from TSS; gene body – +1000 bp from TSS until the end of the coding sequence; downstream – from the end of the coding sequence to +5000 bp. Gene activity was scored based on RPKM and genes with RPKM>0.6 were considered as expressed. (PDF) [file pgen.1004153.s009.pdf]

Zuin et al., Table S3

Position of binding sites relative to genes

Around each gene four regions were designed:

upstream: -5000 to -1000 bp from transcription start site
promoter: -1000 to +1000 bp around transcription start site
gene body: +1000 bp from transcription start site until end of the coding sequence
downstream: end of the coding sequence + 5000 bp

Binding sites of NIPBL, CTCF, SMC1A, SMC3 and RNA Pol II were sorted in these categories. The respective genes were scored for their activity calculating RPKM (reads mapping to the genome per kilobase of transcript per million reads sequenced) Genes with RPKM values >0.6 were scored as expressed.

		NIPBL			
		Position around the gene			
		upstream	promoter	gene body	downstream
Expression	no	3	6	1	2
	yes	24	906	48	26
	**Total sites:**	1138	**Sites around genes:**		1016

		CTCF			
		Position around the gene			
		upstream	promoter	gene body	downstream
Expression	no	415	333	1169	420
	yes	1589	4405	11861	1551
	**Total sites:**	35668	**Sites around genes:**		21743

		SMC1A			
		Position around the gene			
		upstream	promoter	gene body	downstream
Expression	no	301	227	835	328
	yes	1200	2761	9545	1304
	**Total sites:**	29441	**Sites around genes:**		16501

		SMC3			
		Position around the gene			
		upstream	promoter	gene body	downstream
Expression	no	214	152	448	221
	yes	1094	2686	7727	1115
	**Total sites:**	22572	**Sites around genes:**		13657

		RNA Pol II			
		Position around the gene			
		upstream	promoter	gene body	downstream
Expression	no	18	40	7	6
	yes	319	7150	1205	403
	**Total sites:**	9879	**Sites around genes:**		9148
