# Supplementary material for: A Cohesin-Independent Role for NIPBL at Promoters Provides Insights in CdLS
Source: PLoS Genet. 2014 Feb 13;10(2):e1004153. doi: 10.1371/journal.pgen.1004153 (PMC3923681; doi:10.1371/journal.pgen.1004153)
Supplement: Table S4 — Classification of promoters and expression status of genes bound by NIPBL, cohesin (SMC1A and SMC3), RNA Pol II and CTCF. The region −1000 to + 1000 bp from TSS was considered as promoter region. (PDF) [file pgen.1004153.s010.pdf]

Zuin et al., Table S4

Classification of promoters and expression status of genes bound by NIPBL[1], cohesin (SMC1A and SMC3), RNA Pol II and CTCF
([1] the identical definition of the NIPBL-gene association as in Table S3)

	NIPBL			
		CpG island in promoter		
		no	yes	**Total:**
Expression	no	27	36	63
	yes	85	868	953
	**Total sites:**	1138	**Sites around genes:**	1016

	SMC1A			
		CpG island in promoter		
		no	yes	**Total:**
Expression	no	3102	2624	5726
	yes	1816	8959	10775
	**Total sites:**	29441	**Sites around genes:**	16501

	SMC3			
		CpG island in promoter		
		no	yes	**Total:**
Expression	no	2132	1724	3856
	yes	1578	8223	9801
	**Total sites:**	22572	**Sites around genes:**	13657

	RNA Pol II			
		CpG island in promoter		
		no	yes	**Total:**
Expression	no	149	197	346
	yes	710	8092	8802
	**Total sites:**	9879	**Sites around genes:**	9148

	CTCF			
		CpG island in promoter		
		no	yes	**Total:**
Expression	no	3894	3348	7242
	yes	2234	12267	14501
	**Total sites:**	35668	**Sites around genes:**	21743
