# Supplementary material for: A Cohesin-Independent Role for NIPBL at Promoters Provides Insights in CdLS
Source: PLoS Genet. 2014 Feb 13;10(2):e1004153. doi: 10.1371/journal.pgen.1004153 (PMC3923681; doi:10.1371/journal.pgen.1004153)
Supplement: Table S5 — Functional annotation of genes with NIPBL binding sites in HB2 cells by IPA analysis. (PDF) [file pgen.1004153.s011.pdf]

Zuin et al., Table S5

Molecular function of genes with NIPBL binding sites in HB2 cells identified by IPA analysis (IPA version 9.0, IGENUITY SYSTEMS)

Category	Genes	p-value
Cell Cycle	105	2,17E-08-3,56E-02
Gene Expression	147	4,83E-08-3,59E-02
Organismal Development	84	1,68E-07-3,64E-02
RNA Post-Transcriptional Modification	30	7,3E-07-1,43E-02
Cell Death	165	1,6E-06-3,66E-02
Cellular Growth and Proliferation	108	3,14E-05-2,9E-02
Infectious Disease	62	3,42E-05-2,9E-02
Embryonic Development	54	6,9E-05-3,46E-02
Cardiovascular System Development and Function	18	6,93E-05-3,35E-02
Cancer	220	7,32E-05-3,74E-02
Developmental Disorder	39	1,09E-04-3,79E-02
Hematological System Development and Function	9	1,3E-04-1,63E-02
Hematopoiesis	13	1,3E-04-2,07E-02
Renal and Urological Disease	33	1,55E-04-3,74E-02
Dermatological Diseases and Conditions	27	1,96E-04-3,79E-02
