# Supplementary material for: A Cohesin-Independent Role for NIPBL at Promoters Provides Insights in CdLS
Source: PLoS Genet. 2014 Feb 13;10(2):e1004153. doi: 10.1371/journal.pgen.1004153 (PMC3923681; doi:10.1371/journal.pgen.1004153)
Supplement: Table S6 — Primers used for ChIP/qPCR and RT-PCR/qPCR. (PDF) [file pgen.1004153.s012.pdf]

Zuin et al., Table S6

Primers used for ChIP/qPCR and RT-PCR/qPCR

ChIP/qPCR primer

Name	forward	reverse	Position (hg18)
cohesin binding sites			
**chr22_11**	GGCTCAGGACAGAAGTGACC	AGGTCGGCAGAGGCTCC	chr22:31255080-31255266
**chr22_12**	ACATGTGGCCAGCTCAGG	GGCGCTATAAGCCAGAGAAC	chr22:31522929-31523153
**STAT3_3**	CCTCCCTTACCGCTGATGTC	CTAAGCCTCCAGGCACCTTC	chr17:37728538-37728637
**KCNQ1_1**	AGGACCCGCATGAGGGATTC	CCGCATATCCCTGGTTCAGC	chr11:2510878-2511089
**KCNQ1_2**	AAAGGTGTTTGCAGCCCTTG	AACATCGTGCTCTCGGAATC	chr11:2510713-2510912
NIPBL binding sites			
**OSBP**	GCTGCTGTTTCCGCCATTCATTTC	GCTGATACCAACCACCAATCCATGAG	chr11:59140122-59140236
**HMGCS1**	AATGACGGAGCTGCGAGATAC	TTGCTAGGCAACCTGACAGAC	chr5:43349567-43349749
**DUSP10**	TCGGCTTCATTGATCTCCAG	AGAGCAGCTTCGGATAAACC	chr1:219982022-219982252
**STAT3_1**	CCTCCCTTACCGCTGATGTC	CTAAGCCTCCAGGCACCTTC	chr17:37728538-37728637
**GPR108**	GGAGAGCCGATAACGCTTAAC	GCCGGATATCAGATCCATGAC	chr19:6688766-6688880
**GLCCI1**	CACTTACCTCAAGGCTGTCAAC	GCGAGCATTTCCATTGGCTTAG	chr7:7974693-7974884
**MYH9**	GCAGACGTATTGGCCTGTGG	GCCGCCTCCTGATTGGATTC	chr22:35114119-35114326
**STAT3_1**	CCGGAAACCGCTGAATTAC	TAGCTGCTCTCCTCATTGG	chr17:37794031-37794204
**ARTS1**	TAGCGTTGCGAGGGTTAGG	GCACTTTGCCACGACAGAG	chr5:96169763-96169976
**ZNF695**	TCTGGCTGCACGCCTGATTG	GGTCGAAGTGCCGTGAGGAATG	chr1:245238016-245238199
**TSPAN31**	ATGCGGGTTGATTGGCATGCAG	AGGCCAGCCAAACCCAATACTC	chr12:56424994-56425106
**LMBR1L**	CTCTCCAGGAGCCAATGAGTTC	CGCGCTCACGTTTCAATG	chr12:47,790,854-47,790,999
control			
**AMY**	TCTGCTGGGCTCAGTATTCCTC	TGTTGCCCAAGCTTCACGTAG	chr1:104000510-104000730
Repeats			
**LSU_2**	CAGGTGCAGATGTTGGTGGTAG	GAACCGGACTCCCTTTCGATTG	chr1:107914874-107915060
**LSU_8**	GCCGCCACAAGCCAGTTATC	AACCTCCCGTGGAGCAGAAG	chr1:91625487-91625643
**LSU_9**	ACTCAGGATTGGCGTCTTCG	AGCAGTTGAACACGGGTCAG	chr2:229753693-229753871
**LSU_10**	TGCGGTAACGTGACCGATCC	TTTCAAGGGCCAGCGAGAGC	chr5:71182503-71182673
**LSU_11**	GCTCAACAGGGTCTTCTTTCC	AGCCAAATGCCTCGTCATC	chr8:70764913-70765053
**SSU_5**	GCTGGATAGCTGGATAGCTGTC	GAAGGGCAACTGCTTGGAATG	chr15:86163096-86163239
**SSU_6**	GCCGGACTTATCAAGGCAAAC	ACAGGTCTGTGACGCTCATAG	chr5:174474329-174474548
**SSU_7**	GTAGGGCAAGTCTGGTGCCAAC	CTGCTCCCGAGATCCAACTACG	chr7:80800911-80801011

RT-PCR/qPCR primer

Name	forward	reverse	Gene
**TC27**	CATGGTGTCAGAGCACTGAAG	GGTGACTATGCAGGATGATGG	ARTS1
**TC25**	AGCGGAGTTTGTATAGGG	GTTTGGCTGTCTTCTCTG	ZNF695
**TC30**	AGGCGAACCTCCTCTTTGG	GACGCAGAACGCTGATGTG	GLCCI1
**TC31**	TGTCCAGCATCCACATCATC	TAGCCAGACATGAGCAAGAG	TSPAN31
**TC34**	GGAATCCTGCTGCATCAC	TCGTCCTCCTCTTCTTCC	BBX
**TC41**	AAGCAGTGGCTGGTATGAAG	TGAACACAAAGCGCTAGAGG	NIPBL
**TC48**	CACTTGAGCCCAGGAGATTG	TGCCTTTCTGCGACCTTG	MAU2
**TC35**	ACTGGCTACTGCGTACATCC	AGATGCGCCTATCTCTTTCC	NADH
**TC143**	CCAGCAGCGACTCTGAGGAGGAACAAG	GTTTGCTGTGGCCTCCAGCAGAAGGTG	MYC
