# Supplementary material for: A Cohesin-Independent Role for NIPBL at Promoters Provides Insights in CdLS
Source: PLoS Genet. 2014 Feb 13;10(2):e1004153. doi: 10.1371/journal.pgen.1004153 (PMC3923681; doi:10.1371/journal.pgen.1004153)
Supplement: Table S7 — Genes with NIPBL binding sites in a patient-derived lymphoblastoid cells (PT1) found to be differentially expressed in CdLS patients with FDR<0.05 by Liu et al., 2009 (18). (PDF) [file pgen.1004153.s013.pdf]

## Zuin et al., Table S7

### *Genes with NIPBL binding sites in LCL's (PT1) found to be differentially expressed in CdLs patient cells by Liu et al. [18] with FDR < 0.05*

| ID | Entrez Gene Name | Location | Type(s) |
|---|---|---|---|
| **ABHD2** | abhydrolase domain containing 2 | unknown | enzyme |
| **AKAP13** | A kinase (PRKA) anchor protein 13 | Cytoplasm | other |
| **ANKRD11** | ankyrin repeat domain 11 | Nucleus | other |
| **ARRB2** | arrestin, beta 2 | Cytoplasm | other |
| **ATP6V1E1** | ATPase, H+ transporting, lysosomal 31kDa, V1 subunit E1 | Cytoplasm | transporter |
| **ATPIF1** | ATPase inhibitory factor 1 | Cytoplasm | other |
| **BBX** | bobby sox homolog (Drosophila) | Nucleus | other |
| **BUB3** | budding uninhibited by benzimidazoles 3 homolog (yeast) | Nucleus | other |
| **CAT** | catalase | Cytoplasm | enzyme |
| **CDKN3** | cyclin-dependent kinase inhibitor 3 | Nucleus | phosphatase |
| **CDR2** | cerebellar degeneration-related protein 2, 62kDa | Cytoplasm | other |
| **CHAF1A** | chromatin assembly factor 1, subunit A (p150) | Nucleus | other |
| **COPZ1** | coatomer protein complex, subunit zeta 1 | Cytoplasm | transporter |
| **COTL1** | coactosin-like 1 (Dictyostelium) | Cytoplasm | other |
| **CREB3L2** | cAMP responsive element binding protein 3-like 2 | Nucleus | other |
| **CS** | citrate synthase | Cytoplasm | enzyme |
| **DCTN4** | dynactin 4 (p62) | Nucleus | other |
| **DDIT3** | DNA-damage-inducible transcript 3 | Nucleus | transcription regulator |
| **DYNLRB1** | dynein, light chain, roadblock-type 1 | Cytoplasm | other |
| **EBP** | emopamil binding protein (sterol isomerase) | Cytoplasm | enzyme |
| **EIF1** | eukaryotic translation initiation factor 1 | Cytoplasm | translation regulator |
| **ELMO2** | engulfment and cell motility 2 | Cytoplasm | other |
| **EXOC4** | exocyst complex component 4 | Cytoplasm | transporter |
| **F11R** | F11 receptor | Plasma Membrane | other |
| **FBXO8** | F-box protein 8 | Cytoplasm | other |
| **GLCCI1** | glucocorticoid induced transcript 1 | Cytoplasm | other |
| **GLS** | glutaminase | Cytoplasm | enzyme |
| **GOLGB1** | golgin B1 | Cytoplasm | other |
| **GTPBP3** | GTP binding protein 3 (mitochondrial) | Cytoplasm | enzyme |
| **HMGCS1** | 3-hydroxy-3-methylglutaryl-CoA synthase 1 (soluble) | Cytoplasm | enzyme |
| **INTS3** | integrator complex subunit 3 | Nucleus | other |
| **ITFG2** | integrin alpha FG-GAP repeat containing 2 | unknown | other |
| **JMJD2B** | lysine (K)-specific demethylase 4B | unknown | other |
| **KHSRP** | KH-type splicing regulatory protein | Nucleus | enzyme |
| **LAPTM4A** | lysosomal protein transmembrane 4 alpha | Cytoplasm | other |
| **LAS1L** | LAS1-like (S. cerevisiae) | Nucleus | other |
| **LRRC28** | leucine rich repeat containing 28 | unknown | other |
| **MCM3** | minichromosome maintenance complex component 3 | Nucleus | enzyme |
| **MCM5** | minichromosome maintenance complex component 5 | Nucleus | enzyme |
| **MSH2** | mutS homolog 2, colon cancer, nonpolyposis type 1 (E. coli) | Nucleus | enzyme |
| **MTMR4** | myotubularin related protein 4 | Cytoplasm | phosphatase |
| **NBR1** | neighbor of BRCA1 gene 1 | Cytoplasm | other |
| **NCAPD3** | non-SMC condensin II complex, subunit D3 | Nucleus | other |
| **NFE2L1** | nuclear factor (erythroid-derived 2)-like 1 | Nucleus | transcription regulator |
| **NGRN** | neugrin, neurite outgrowth associated | Nucleus | other |

## Zuin et al., Table S7 continued

| ID | Entrez Gene Name | Location | Type(s) |
|---|---|---|---|
| **NR2C2** | nuclear receptor subfamily 2, group C, member 2 | Nucleus | ligand-dependent nuclear receptor |
| **NUP98** | nucleoporin 98kDa | Nucleus | transporter |
| **ORC2L** | origin recognition complex, subunit 2 | Nucleus | other |
| **OSBPL3** | oxysterol binding protein-like 3 | Cytoplasm | other |
| **PBXIP1** | pre-B-cell leukemia homeobox interacting protein 1 | Nucleus | transcription regulator |
| **PCCB** | propionyl CoA carboxylase, beta polypeptide | Cytoplasm | enzyme |
| **PHPT1** | phosphohistidine phosphatase 1 | Cytoplasm | phosphatase |
| **PIAS3** | protein inhibitor of activated STAT, 3 | Nucleus | transcription regulator |
| **PJA2** | praja ring finger 2 | Cytoplasm | enzyme |
| **PLCG1** | phospholipase C, gamma 1 | Cytoplasm | enzyme |
| **PNN** | pinin, desmosome associated protein | Plasma Membrane | other |
| **PPP3CA** | protein phosphatase 3, catalytic subunit, alpha isozyme | Cytoplasm | phosphatase |
| **PTMA** | prothymosin, alpha | Nucleus | other |
| **RAB5B** | RAB5B, member RAS oncogene family | Cytoplasm | enzyme |
| **RFWD3** | ring finger and WD repeat domain 3 | Nucleus | enzyme |
| **RPL17** | ribosomal protein L17 | Cytoplasm | other |
| **RPS6** | ribosomal protein S6 | Cytoplasm | other |
| **RUVBL1** | RuvB-like 1 (E. coli) | Nucleus | transcription regulator |
| **SEC61G** | Sec61 gamma subunit | Cytoplasm | transporter |
| **SFXN5** | sideroflexin 5 | Cytoplasm | transporter |
| **SLC25A37** | solute carrier family 25, member 37 | Cytoplasm | transporter |
| **SLC25A42** | solute carrier family 25, member 42 | Cytoplasm | other |
| **SLC39A3** | solute carrier family 39 (zinc transporter), member 3 | Plasma Membrane | transporter |
| **SNRPA** | small nuclear ribonucleoprotein polypeptide A | Nucleus | other |
| **SORT1** | sortilin 1 | Cytoplasm | transporter |
| **STAT3** | signal transducer and activator of transcription 3 (acute-phase respon | Nucleus | transcription regulator |
| **STT3B** | STT3, subunit of the oligosaccharyltransferase complex, homolog B ( | Cytoplasm | enzyme |
| **TMEM9** | transmembrane protein 9 | Cytoplasm | other |
| **TSPAN31** | tetraspanin 31 | Plasma Membrane | other |
| **UBR1** | ubiquitin protein ligase E3 component n-recognin 1 | Cytoplasm | enzyme |
| **UGP2** | UDP-glucose pyrophosphorylase 2 | Cytoplasm | enzyme |
| **UTP6** | UTP6, small subunit (SSU) processome component, homolog (yeast) | Nucleus | other |
| **WDR74** | WD repeat domain 74 | Nucleus | other |
| **YBX1** | Y box binding protein 1 | Nucleus | transcription regulator |
| **ZFP91** | zinc finger protein 91 homolog (mouse) | Nucleus | transcription regulator |
| **ZNF141** | zinc finger protein 141 | Nucleus | transcription regulator |
| **ZNF211** | zinc finger protein 211 | Nucleus | transcription regulator |
| **ZNF671** | zinc finger protein 671 | Nucleus | other |
| **ZNF691** | zinc finger protein 691 | Nucleus | other |
| **ZNF695** | zinc finger protein 695 | Nucleus | other |
| **ZNF720** | zinc finger protein 720 | unknown | other |
| **ZNF91** | zinc finger protein 91 | Nucleus | transcription regulator |
