# Supplementary material for: A Cohesin-Independent Role for NIPBL at Promoters Provides Insights in CdLS
Source: PLoS Genet. 2014 Feb 13;10(2):e1004153. doi: 10.1371/journal.pgen.1004153 (PMC3923681; doi:10.1371/journal.pgen.1004153)
Supplement: Table S8 — NIPBL ChIP signals in HB2 cells on different repeat classes. RPKM measure (reads per kilobase per 10 million reads) was calculated similar to the RNA-seq analyses (56) and an enrichment compared to the input material (control) was calculated. (PDF) [file pgen.1004153.s014.pdf]

Zuin et al., Table S 8

NIPBL ChIP signals in HB2 cells on different repeat classes.
RPKM measure (reads per kilobase per 10 million reads) was calculated similar to the RNA-seq analyses [56] and an enrichment compared to the input material (control) was calculated.

class	length (bp)	% of total genomic sequence	ChIP signal (RPKM) NIPBL ChIP	ChIP signal (RPKM) control	Enrichment
**SINE-Alu**	306745172	9.91	1.68	1.56	**1.08**
**SINE-MIR**	84349685	2.72	4.75	4.97	**0.96**
**SINE-Other**	571757	0.02	5.94	5.76	**1.03**
**LINE-L1**	512445461	16.55	2.08	1.96	**1.07**
**LINE-L2**	103990071	3.36	5.08	5.18	**0.98**
**LINE-CR1**	10860002	0.35	4.55	4.48	**1.02**
**LINE-RTE**	3643770	0.12	4.08	3.79	**1.08**
**LINE-Other**	201745	0.01	4.89	4.75	**1.03**
**LTR-ERVL-MaLR**	110849894	3.58	4	3.88	**1.03**
**LTR-ERVL**	56136298	1.81	4.86	4.81	**1.01**
**LTR-ERV1**	83494001	2.7	3.2	3.06	**1.04**
**LTR-ERVK**	8842596	0.29	1.94	1.72	**1.13**
**LTR-Gypsy**	2301837	0.07	5.28	5.15	**1.03**
**LTR-ERV**	191609	0.01	5.36	4.77	**1.12**
**DNA-TcMar-Tigger**	33986503	1.1	3.15	3.03	**1.04**
**DNA-TcMar-Mariner**	2830187	0.09	3.29	3.11	**1.06**
**DNA-TcMar-Tc2**	1669939	0.05	3.24	3.08	**1.05**
**DNA-TcMar**	318232	0.01	3.82	3.53	**1.08**
**DNA-hAT-Charlie**	45014464	1.45	4.17	4.03	**1.04**
**DNA-hAT-Blackjack**	3419498	0.11	4.31	4.22	**1.02**
**DNA-hAT-Tip100**	6614899	0.21	4.35	4.32	**1.01**
**DNA-hAT**	1687099	0.05	4.11	4.09	**1.01**
**DNA-Other**	2734962	0.09	4.06	3.79	**1.07**
**Satellite-acro**	31038	0	14.04	11.47	**1.22**
**Satellite-centr**	8243430	0.27	10.45	9.17	**1.14**
**Satellite-telo**	254147	0.01	3.2	4.27	**0.75**
**Satellite**	4025177	0.13	18.3	17.69	**1.03**
**rRNA**	175474	0.01	265.35	19.96	**13.3**
LSU-rRNA_Hsa	*68641*	*0.002*	*442.08*	*28.92*	*15.28*
SSU-rRNA_Hsa	*16619*	*0.001*	*947.81*	*66.34*	*14.29*
5S	*90214*	*0.003*	*5.15*	*4.59*	*1.12*
**tRNA**	102988	0	8.09	13.2	**0.61**
**scRNA**	122042	0	3.5	3.5	**1**
**snRNA**	337551	0.01	5.57	4.13	**1.35**
**srpRNA**	263136	0.01	4.09	4.24	**0.96**
**RNA**	118748	0	4.73	5.74	**0.82**
**Low_complexity**	16922589	0.55	2.79	2.35	**1.19**
**Simple_repeat**	26087578	0.84	2.78	3.15	**0.88**
**Other repeat**	6109487	0.2	1.85	1.83	**1.01**
